# Clinicogenomic factors of biotherapy immunogenicity in autoimmune disease: A prospective multicohort study of the ABIRISK consortium

**DOI:** 10.1371/journal.pmed.1003348

**Published:** 2020-10-30

**Authors:** Signe Hässler, Delphine Bachelet, Julianne Duhaze, Natacha Szely, Aude Gleizes, Salima Hacein-Bey Abina, Orhan Aktas, Michael Auer, Jerôme Avouac, Mary Birchler, Yoram Bouhnik, Olivier Brocq, Dorothea Buck-Martin, Guillaume Cadiot, Franck Carbonnel, Yehuda Chowers, Manuel Comabella, Tobias Derfuss, Niek De Vries, Naoimh Donnellan, Abiba Doukani, Michael Guger, Hans-Peter Hartung, Eva Kubala Havrdova, Bernhard Hemmer, Tom Huizinga, Kathleen Ingenhoven, Poul Erik Hyldgaard-Jensen, Elizabeth C. Jury, Michael Khalil, Bernd Kieseier, Anna Laurén, Raija Lindberg, Amy Loercher, Enrico Maggi, Jessica Manson, Claudia Mauri, Badreddine Mohand Oumoussa, Xavier Montalban, Maria Nachury, Petra Nytrova, Christophe Richez, Malin Ryner, Finn Sellebjerg, Claudia Sievers, Dan Sikkema, Martin Soubrier, Sophie Tourdot, Caroline Trang, Alessandra Vultaggio, Clemens Warnke, Sebastian Spindeldreher, Pierre Dönnes, Timothy P. Hickling, Agnès Hincelin Mery, Matthieu Allez, Florian Deisenhammer, Anna Fogdell-Hahn, Xavier Mariette, Marc Pallardy, Philippe Broët, ABIRISK consortium

**Affiliations:** 1 CESP, INSERM UMR 1018, Faculty of Medicine, Paris-Sud University, UVSQ, Paris-Saclay University, Villejuif, France; 2 Sorbonne Université, INSERM UMR 959, Immunology-Immunopathology-Immunotherapy (i3), Paris, France; 3 AP-HP, Hôpital Pitié-Salpêtrière, Biotherapy (CIC-BTi), Paris, France; 4 Department of Biostatistical Epidemiology and Clinical Research, Hôpital Bichat, Assistance Publique-Hôpitaux de Paris AP-HP.Nord, INSERM CIC-EC 1425, Paris, France; 5 CHU Ste-Justine Research Center, Montreal, Canada; 6 INSERM UMR 996, Faculty of Pharmacy, Paris-Sud University, Paris-Saclay University, Châtenay-Malabry, France; 7 Clinical Immunology Laboratory, AP-HP, Le Kremlin-Bicêtre Hospital, Paris-Sud University Hospitals, Le Kremlin-Bicêtre, France; 8 UTCBS, CNRS UMR 8258, INSERM U1022, Faculty of Pharmacy, Paris-Descartes-Sorbonne-Cite University, Paris, France; 9 University of Düsseldorf, Medical Faculty, Department of Neurology, Düsseldorf, Germany; 10 Innsbruck Medical University, Department of Neurology, Innsbruck, Austria; 11 Paris University, Paris Descartes University, INSERM U1016, Paris, France; 12 Rheumatology department, Cochin Hospital, AP-HP.CUP, Paris, France; 13 GlaxoSmithKline, Clinical Immunology–Biopharm, Collegeville, Pennsylvania, United States of America; 14 AP-HP, Hôpital Beaujon, Paris, France; 15 GETAID, Paris, France; 16 Princess Grace Hospital, Rheumatology, Monaco; 17 Department of Neurology, Technische Universität München, Munich, Germany; 18 Service d'hépato-gastroentérologie, University Hospital of Reims, Reims, France; 19 Department of Gastroenterology, AP-HP, Hôpital Kremlin-Bicêtre, France; 20 Department of Gastroenterology, Rambam Health Care Campus, Haifa, Israel; Bruce Rappaport School of Medicine, Technion Israel Institute of Technology, Haifa, Israel; Clinical Research Institute, Rambam Health Care Campus, Haifa, Israel; 21 Servei de Neurologia-Neuroimmunologia, Centre d’Esclerosi Múltiple de Catalunya (Cemcat). Institut de Recerca Vall d’Hebron (VHIR). Hospital Universitari Vall d’Hebron, Universitat Autònoma de Barcelona, Barcelona, Spain; 22 Departments of Biomedicine and Neurology, University Hospital Basel and University of Basel, Basel, Switzerland; 23 Rheumatology & Clinical Immunology, Amsterdam UMC | AMC, University of Amsterdam, Amsterdam, the Netherlands; 24 Ipsen Biopharm Ltd, Berkshire, United Kingdom; 25 Sorbonne Université, Inserm, UMS Production et Analyse des données en Sciences de la vie et en Santé, UMS 37 PASS, Plateforme Post-génomique de la Pitié-Salpêtrière, P3S, Paris, France; 26 Clinic for Neurology 2, Med Campus III, Kepler University Hospital GmbH, Linz, Austria; 27 Department of Neurology and Center of Clinical Neuroscience, First Faculty of Medicine, Charles University and General University Hospital, Prague, Czech Republic; 28 Munich Cluster for Systems Neurology (SyNergy), Munich, Germany; 29 Department of Rheumatology, Leiden University Medical Center, Leiden, the Netherlands; 30 Danish Multiple Sclerosis Center, Department of Neurology, Rigshospitalet, University of Copenhagen, Copenhagen, Denmark; 31 Centre for Rheumatology Research, University College London, London, United Kingdom; 32 Department of Neurology, Medical University of Graz, Austria; 33 Svar Life Science, Malmö, Sweden; 34 Dipartimento di Medicina Sperimentale e Clínica, Università di Firenze, Firenze, Italy; 35 Immunology Area of Bambino Gesù Pediatric Hospital, IRCCS, Rome, Italy; 36 Department of Rheumatology, University College London Hospital, London, United Kingdom; 37 Center for Multiple Sclerosis, St. Michael's Hospital, University of Toronto, Toronto, Canada; 38 University hospital of Lille, Maladies de l'appareil digestif, Lille, France; 39 Rheumatology Department, CHU de Bordeaux-GH Pellegrin, Bordeaux, France; 40 UMR CNRS 5164, Bordeaux University, Bordeaux, France; 41 Department of Clinical Neuroscience, Karolinska Institutet, Stockholm, Sweden; 42 Current address: Quanterix Corporation, Billerica, Massachusetts, United States of America; 43 Rheumatology, University Hospital of Clermont Ferrand, Clermont Ferrand, France; 44 Institut des maladies de l'Appareil Digestif, University Hospital of Nantes, Nantes, France; 45 Department of Neurology, University Hospital Köln, Köln, Germany; 46 Drug Metabolism Pharmacokinetics-Biologics, Novartis Institutes for Biomedical Research, Basel, Switzerland; 47 Integrated Biologix GmbH, Basel, Switzerland; 48 SciCross AB, Skövde, Sweden; 49 BioMedicine Design, Pfizer, Inc., Andover, Massachusetts, United States of America; 50 Sanofi, Chilly-Mazarin, France; 51 Department of Gastroenterology, Hôpital Saint-Louis, AP-HP, Université Paris-Diderot, Paris, France; 52 Centre for Immunology of Viral Infections and Autoimmune Diseases, INSERM UMR 1184, Université Paris-Saclay, AP-HP.Université Paris-Saclay, Le Kremlin-Bicêtre, France; 53 AP-HP, Paris-Sud University Hospitals, Paul Brousse Hospital, Villejuif, France; Arthritis Research UK Epidemiology Unit, UNITED KINGDOM

## Abstract

**Background:**

Biopharmaceutical products (BPs) are widely used to treat autoimmune diseases, but immunogenicity limits their efficacy for an important proportion of patients. Our knowledge of patient-related factors influencing the occurrence of antidrug antibodies (ADAs) is still limited.

**Methods and findings:**

The European consortium ABIRISK (Anti-Biopharmaceutical Immunization: prediction and analysis of clinical relevance to minimize the RISK) conducted a clinical and genomic multicohort prospective study of 560 patients with multiple sclerosis (MS, n = 147), rheumatoid arthritis (RA, n = 229), Crohn’s disease (n = 148), or ulcerative colitis (n = 36) treated with 8 different biopharmaceuticals (etanercept, n = 84; infliximab, n = 101; adalimumab, n = 153; interferon [IFN]-beta-1a intramuscularly [IM], n = 38; IFN-beta-1a subcutaneously [SC], n = 68; IFN-beta-1b SC, n = 41; rituximab, n = 31; tocilizumab, n = 44) and followed during the first 12 months of therapy for time to ADA development. From the bioclinical data collected, we explored the relationships between patient-related factors and the occurrence of ADAs. Both baseline and time-dependent factors such as concomitant medications were analyzed using Cox proportional hazard regression models. Mean age and disease duration were 35.1 and 0.85 years, respectively, for MS; 54.2 and 3.17 years for RA; and 36.9 and 3.69 years for inflammatory bowel diseases (IBDs). In a multivariate Cox regression model including each of the clinical and genetic factors mentioned hereafter, among the clinical factors, immunosuppressants (adjusted hazard ratio [aHR] = 0.408 [95% confidence interval (CI) 0.253–0.657], p < 0.001) and antibiotics (aHR = 0.121 [0.0437–0.333], p < 0.0001) were independently negatively associated with time to ADA development, whereas infections during the study (aHR = 2.757 [1.616–4.704], p < 0.001) and tobacco smoking (aHR = 2.150 [1.319–3.503], p < 0.01) were positively associated. 351,824 Single-Nucleotide Polymorphisms (SNPs) and 38 imputed Human Leukocyte Antigen (HLA) alleles were analyzed through a genome-wide association study. We found that the HLA-DQA1*05 allele significantly increased the rate of immunogenicity (aHR = 3.9 [1.923–5.976], p < 0.0001 for the homozygotes). Among the 6 genetic variants selected at a 20% false discovery rate (FDR) threshold, the minor allele of rs10508884, which is situated in an intron of the *CXCL12* gene, increased the rate of immunogenicity (aHR = 3.804 [2.139–6.764], p < 1 × 10^−5^ for patients homozygous for the minor allele) and was chosen for validation through a CXCL12 protein enzyme-linked immunosorbent assay (ELISA) on patient serum at baseline before therapy start. CXCL12 protein levels were higher for patients homozygous for the minor allele carrying higher ADA risk (mean: 2,693 pg/ml) than for the other genotypes (mean: 2,317 pg/ml; p = 0.014), and patients with CXCL12 levels above the median in serum were more prone to develop ADAs (aHR = 2.329 [1.106–4.90], p = 0.026). A limitation of the study is the lack of replication; therefore, other studies are required to confirm our findings.

**Conclusion:**

In our study, we found that immunosuppressants and antibiotics were associated with decreased risk of ADA development, whereas tobacco smoking and infections during the study were associated with increased risk. We found that the HLA-DQA1*05 allele was associated with an increased rate of immunogenicity. Moreover, our results suggest a relationship between CXCL12 production and ADA development independent of the disease, which is consistent with its known function in affinity maturation of antibodies and plasma cell survival. Our findings may help physicians in the management of patients receiving biotherapies.

## Abstract 2

In a multicohort prospective study of patients from 12 countries, Signe Hässler and colleagues investigate clinical and genetic factors associated with development of anti-biopharmaceutical drug antibodies in patients with autoimmune diseases.

## Author summary

**Why was this study done?:**

- Biopharmaceutical products such as monoclonal antibodies are widely used to treat autoimmune diseases.
- Biopharmaceutical products may induce the development of antidrug antibodies, which often lead to therapy failure.
- Patient-related factors that influence the development of antidrug antibodies need to be characterized.

**What did the researchers do and find?:**

- We set up a European multicohort prospective study on 4 autoimmune diseases (multiple sclerosis, rheumatoid arthritis, Crohn’s disease, and ulcerative colitis) treated with 8 different biopharmaceutical products.
- We collected demographic and clinical data and tested antidrug antibodies in longitudinal serum samples from 560 patients. For 457 patients who gave consent, we also collected genetic data.
- We identified antibiotics and immunosuppressants as negatively associated risk factors and heavy smoking, infections during the study, the HLA-DQA1*05 allele and a minor variant in the *CXCL12* chemokine gene associated with increased protein expression as risk factors of antidrug antibody development.

**What do these findings mean?:**

- Our findings suggest that the combination of immunosuppressant and biopharmaceutical therapy could be associated with decreased risk of antidrug antibody occurrence, which has implications for rheumatoid arthritis and inflammatory bowel diseases, for which immunosuppressants are often, but not always, given together with biopharmaceuticals.
- Patients heterozygotes or homozygotes for the HLA-DQA1*05 allele may have an increased risk of antidrug antibody occurrence associated with biopharmaceutical therapy.
- The small study size warrants a validation through independent studies, in particular for the genetic findings.

## Introduction

Biopharmaceutical products (BPs) represent a fast-growing class of therapeutics that includes, but is not limited to, replacement factors for abnormal or deficient proteins, cytokines and growth factors modulating biological functions, monoclonal antibodies targeting components of disease pathways, fusion proteins, and protein–drug conjugates. The current use of these BPs in the clinic represents a major improvement in the treatment of many severe autoimmune diseases and in cancer.

A drawback is that patients can be immunized to these BPs, leading to the formation of antibodies against the drug. The consequences range from appearance of low-titer antidrug antibodies (ADAs) without any clinical significance to severe loss of efficacy by either blocking the drug or enhancing drug clearance. The mechanisms leading to immunogenicity against the biotherapy can either be patient-related (genetic background, immunological status, prior exposure, prior disease, coadministered drugs) or treatment-related (drug characteristics and formulations, route, dose, frequency of administration), but their relative contributions to the development of ADAs are not fully understood and remain to be deciphered. Major efforts for minimizing product-related factors involved in immunogenicity have been made; however, there is still an urgent need to identify patient-related factors that may provide a basis for stratified/personalized therapeutic approaches. Among patient-related risk factors for immunogenicity, the genetic diversity in immune regulatory genes is likely to play a major role in the development of ADAs [1–3].

In this context, the Innovative Medicine Initiative-funded ABIRISK consortium (Anti-Biopharmaceutical Immunization: prediction and analysis of clinical relevance to minimize the RISK) [4,5] was created to provide an integrated approach to BP immunization, bringing together a large network of researchers from basic to clinical immunology. The main goals of this consortium were to shed new light on BP immunogenicity, identify new ways to produce safer biopharmaceuticals, and generate tools to predict how individual patients are likely to respond to BPs. For this latter objective, a real-world observational prospective multicenter cohort of patients suffering from various autoimmune diseases (multiple sclerosis [MS], rheumatoid arthritis [RA], and inflammatory bowel diseases [IBDs]) was established. All participating patients were naive for the biotherapies they were given during the study, which included tumor necrosis factor (TNF) inhibitors, interferon (IFN)-beta, and anti-CD20 (Cluster of Differentiation 20) and anti-interleukin (IL)6 receptor monoclonal antibodies. The main objective was to identify baseline bioclinical factors and genetic markers associated with the BP immunization that were able to predict the occurrence of ADAs within the first year of treatment across the different autoimmune diseases and BPs using ADA assays that were developed and validated using current recommendations used by the pharmaceutical industry [6].

The findings of this work rely upon a multicohort analysis that combines information from multiple autoimmune diseases and drugs. The rationale for considering such a multidisease analysis is that even though these autoimmune diseases encompass a broad range of phenotypic manifestations, the similar immunogenic response to various BPs suggests that the pathways involved probably share some clinical factors and genetic variants. This integrative strategy, which borrows information across several therapies and autoimmune diseases, is likely to provide significant gain in power in finding general patient-related risk factors not bound to specific therapies or autoimmune diseases as compared to separate autoimmune disease analyses. This approach could also provide new insights into common immunogenicity mechanisms, together with new opportunities for patient stratification before exposure to a new drug based on his/her genetic makeup.

## Methods

### Study design and patient characteristics

The patients included in the present study were recruited within 3 multicentric prospective studies (ABI-MS-P01, ABI-RA-P01, ABI-IBD-P01) in 12 European and associated countries (Israel) and 50 sites adhering/participating to the ABIRISK EU consortium. All the included patients gave a written informed consent for the clinical study and a second distinct informed consent for the genetic study (in accordance with the Declaration of Helsinki principles). This study is reported as per the Strengthening the Reporting of Observational Studies in Epidemiology (STROBE) guideline (S1 Checklist).

The ethics approvals for the ABI-MS-P01 study (EudraCT Number 2012-005450-30) were obtained from the Medical Ethics Committee of the General University Hospital in Prague (reference 125/12, Evropský grant 1.LF UK-CAGEKID) for Czech Republic; from the institutional committee of Heinrich Heine University, Düsseldorf, Germany (protocol reference 4451) for Düsseldorf; from the Ethikkommission der Fakultät für Medizin der Technischen Universität München, München, Germany (reference 335/13) for München; from the Ethikkommission Nordwest- und Zentralschweiz, Basel (reference 305/13) for Switzerland; from the Ethikkommission der Medizinischen Universität Innsbruck, Innsbruck (reference AN2013-0040 331/2.1) for Austria; from the Comité Ético de Investigación Clínica de l'Hospital Universitari Vall d'Hebrón, Barcelona (reference EPA[AG]66/2013[3866]) for Spain; and from the Stockholm Regional Ethics Committee, Stockholm (reference Dnr. 2013/1034-31/3 and Dnr. 2015/749-32) and Medical Product Agency (MPA) Dnr 5.1-2013-108370 for Sweden.

The ethics approvals for the ABI-RA-P01 study (ClinicalTrials.gov, NCT02116504) were obtained from the Comité de Protection des Personnes Ile de France VII (reference 13–048) for France; from the Medical Ethical Committee of the Academisch Medisch Centrum, Amsterdam (reference 2013–304#B20131074) for the Netherlands; from the Local Ethics Committee of AOU Careggi (reference Protocol Number 2012/0035982) for Italy; and from the NRES Committee London, City & East (reference 14/LO/0506) for the United Kingdom.

The ethics approvals for the ABI-IBD-P01 study were obtained from the Comité de Protection des Personnes Ile de France IV (reference 2013/24) for France; from the Comité d'éthique hospitalo-facultaire universitaire de Liège (reference 2015/55) for Belgium; and from the Helsinki committee of the Rambam Health Care Campus (reference 0075–09) for Israel.

Patients aged 18 years or more who had been prescribed the identified BP for the first time by a physician independently of the study were followed for 12 months from the start of the therapy, during which 7 to 12 visits were performed (specific to each disease study protocol), clinical data were recorded into an electronic Case Report Form (eCRF), and DNA samples and serum samples were collected for genetic analyses and ADA testing, respectively.

*ABI-MS-P01 study*. Patients with MS or Clinically Isolated Syndrome (CIS) diagnosis according to McDonald criteria and who were prescribed IFN-beta-1a intramuscularly (IM), IFN-beta-1a subcutaneously (SC), or IFN-beta-1b SC (original product or biosimilar) were recruited from January 30, 2014 to January 22, 2016. Patients who were not naive to IFN-beta were excluded.

*ABI-RA-P01 study*. Patients with RA diagnosis according to 2010 ACR/EULAR criteria and who were prescribed for the first time a BP among the TNF inhibitors adalimumab, infliximab (original product or biosimilar), etanercept, the anti-CD20 antibody rituximab, or the anti-IL6R antibody tocilizumab (administered SC or intravenously) as first-line or second-line therapy were recruited from March 3, 2014 to June 21, 2016. Patients who were not naive for the biotherapy they were given in the study were excluded.

*ABI-IBD-P01 study*. Patients with Crohn’s disease or ulcerative colitis diagnosis according to ECCO guidelines and who were prescribed adalimumab or infliximab (original product or biosimilar) were recruited from September 11, 2013 to February 15, 2016. Patients who were not naive to TNF inhibitor therapy or who were treated with corticoids more than 30 mg/day over the last 15 days were excluded. All the data from the different sites have been merged with the ADA test results and gathered in a unique database (TranSMART) hosted by the eTRIKS consortium, following standardized data-loading procedures according to CDISC terminology.

Data recorded at the baseline visit included demographic characteristics, vital signs and disease-specific clinical scores, smoking history, familial history of disease, medical and surgical history, vaccines in the year before the study, previous medications, and concomitant medications. Data recorded at each study visit included new vaccines, adverse events, and concomitant medications during the study. At the end of the study, familial history of disease, medical and surgical history, and adverse events were encoded to standardized terms with the MedDRA dictionary, and previous and concomitant medications and vaccines were encoded with the WHODrug dictionary. From the MedDRA classification, System Organ Class (SOC) was used for the analyses and the High-Level Group Term (HLGT) or the Preferred Term (PT) for the most frequent SOC terms. For familial history of the same autoimmune disease, the Lowest-Level Terms (LLTs) were used. From the WHODrug classification, the Anatomic Therapeutic Chemical level 2 (ATC2 subclasses) were retained for the analyses. Exposures with a frequency of at least 5% in each of the 3 disease cohorts were retained for analyses, and during the study, exposures before first ADA detection were treated as time-dependent variables.

### ADA assays

Serum samples for ADA testing were collected at baseline before start of BP therapy and subsequently at each study visit after start of therapy. ADAs were detected by specific validated assays for each BP and analyzed in central ABIRISK laboratories. For IFN-beta, binding antibodies (BAbs) were tested with a previously described bridging enzyme-linked immunosorbent assay (ELISA) at the University of Düsseldorf, Department of Neurology, Düsseldorf, Germany, and Neutralizing antibodies (NAbs) were detected with a cell-based luciferase reporter gene assay in Region Hovedstaden Neuroimmunology laboratory, Department of Neurology, Rigshospitalet, Copenhagen, Denmark. Because of the higher sensitivity of the NAb assay, a serum sample was defined as IFN-beta ADA positive if it was either BAb positive or NAb positive (with an NAb titer equal or higher than 320 U/ml) or positive for both criteria [7–9]. For adalimumab and infliximab and its biosimilars, BAbs were measured with a chemoluminescence drug-tolerant capture ELISA assay using an MSD (MesoScale Discovery) platform at the INSERM UMR 996, Inflammation, Chemokines and Immunopathology laboratory, Châtenay-Malabry, France. Etanercept BAbs were tested with specific bridge-ELISAs (LISA-TRACKER, Theradiag, Croissy-Beaubourg, France) at the Clinical Immunology laboratory of the Kremlin-Bicêtre hospital, France. Rituximab BAbs were tested with a chemoluminescence drug-tolerant capture ELISA assay using an MSD platform at the clinical immunology laboratory of GlaxoSmithKline Research and Development, Upper Merion, PA, USA. Tocilizumab BAbs were tested with a chemoluminescence drug-tolerant capture ELISA assay using an MSD platform at the Svar Life Science laboratory in Malmö, Sweden. The ADA methods had been validated at each laboratory using recommendations from industry approved validation processes using drug-specific positive control (PC) antibodies to validate each assay performance, including sensitivity and drug tolerance [6].

### CXCL12 laboratory test

The CXCL12 baseline serum levels of 108 patients suffering from RA were assessed with a quantitative sandwich ELISA kit (R&D Systems, Minneapolis, MN, USA; sensitivity 47 pg/ml). Analysts were blinded with regard to ADA status of the patients.

### DNA extraction and genotyping

**Fig 1:**
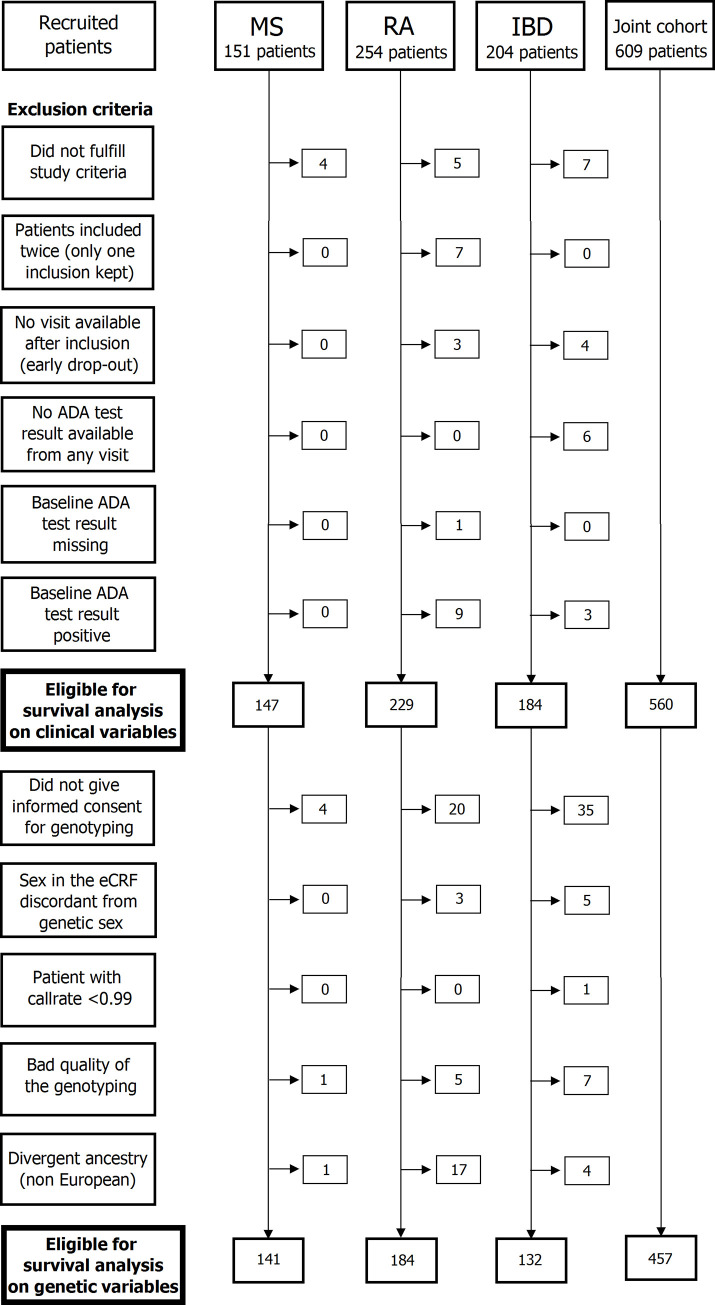
Flow chart of recruited patients. ADA, antidrug antibody; eCRF, electronic Case Report Form; IBD, inflammatory bowel disease; MS, multiple sclerosis; RA, rheumatoid arthritis.

Patients who consented for genetic testing with available high-quality blood DNA samples and valid clinical information were selected for genotyping (Fig 1). Blood for DNA extraction was collected into EDTA tubes and stored at −80°C until purification. DNA was purified using the QIAamp DNA Blood Maxi Kit (Qiagen, Hilden, Germany) according to manufacturer’s instructions. Genotyping was performed at the P3S core facility (Sorbonne University, Paris, France). The genomic DNA concentration was measured by Quant-iT dsDNA Broad range Assay (Thermo Fisher Scientific, Waltham, MA, USA) in a Tecan infinite 200 microplate Reader (TECAN, Männedorf, Switzerland). The DNA integrity was assessed using 1% agarose gel. The DNA polymorphism analysis is based on Illumina BeadArray technology and performed with Infinium OmniExpress-24 v1.2 BeadChip. DNA samples were genotyped according to Infinium HTS Automated Protocol (Illumina, San Diego, CA, USA) starting from 200 ng of double-stranded DNA.

A first quality control check of the genotyping data and genotype calling were performed by Genome Studio software 2011.1 with Genotyping module v1.9 (Illumina). Genotypes are called by comparing the generated data with those in the supplied cluster file. Samples with a call rate (percentage of SNPs genotyped by samples) lower than 95% in the Illumina Cluster were excluded. From this data set, a second quality control check was performed, first on the level of the individual and then on the level of the genetic marker, using the R package “gaston” [10]. Individuals were excluded for ambiguous sex (genotypic sex different from phenotypic sex from the eCRF), abnormal heterozygosity (deviated more than 3 standard deviations [SDs] from the mean heterozygosity of the sample), genotyping completeness less than 99%, and non-European ethnicity admixture detected as outliers from a principal component analyses of a linkage-disequilibrium-pruned data set (with a deviation of at least 6 SDs from the mean of at least one of the first 10 principal components). The quality control left 457 genotyped individuals for the analyses. The initial number of genotyped SNPs was 713,599. Markers were excluded from the analyses if they had a Minor Allele Frequency (MAF) below 5%, if they had a call rate below 99%, or if they deviated significantly from the estimated allele frequencies at Hardy–Weinberg equilibrium (based on a threshold of p < 1 × 10^−5^).

In order to prevent departure from the asymptotic null distribution of the log-rank test related to treatment group imbalance, we performed the genome-wide association analysis only on the variants with an MAF higher than 20%. The final number of SNPs included in the analysis was 351,824 SNPs.

Concerning HLA allele imputation, a statistical imputation of classical *HLA* alleles was performed using SNP data with the HIBAG method, with attribute bagging trained from individuals of European ancestries and based on Infinium OmniExpress 24-chip [11]. Imputed *HLA* loci were *HLA-A*, *B*, *C*; *DRB1*; *DQA1*; *DQB1*; and *DPB1* at 2-digit resolution, and 98 *HLA* markers were imputed. Only 38 HLA alleles with an MAF higher than 5% and that did not deviate significantly from the estimated allele frequencies at Hardy–Weinberg equilibrium (based on a threshold of p < 1 × 10^−5^) were retained for the analysis.

### Statistical analysis

The statistical analysis plan underlying this study is available in S1 Text.

#### Cohort characteristics analysis

Study participants were described by baseline characteristics per disease. Common baseline characteristics and biological samples were compared between patients and diseases using the chi-square test, Fisher’s exact test, and Welch's *t* test when appropriate. A p-value below 0.05 was considered significant.

#### Time-to-event outcomes

To take into account that ADA occurrence is a dynamic event that appears mainly within the 12-month window after the start of therapy, we performed time-to-event analyses. The time to event (ADA positivity) was defined as the period of time from the date of first treatment to the time of first ADA positivity. Patients without ADA occurrence were censored at the date of their last follow-up (drop-out, drug switch) or administrative censoring (12 months).

#### Survival analysis

The search for clinical variables associated with the immunogenicity response was first performed with a Cox proportional hazards regression model. Non-time–dependent variables such as age, disease, sex, tobacco smoking, body mass index (BMI), recent vaccination, familial history of same autoimmune diseases, associated pathology (hypertension, metabolic, neoplastic, nervous, respiratory), past infections, past corticosteroids, and past immunosuppressants were tested. Time-dependent variables such as infections during the study and concomitant medications (immunosuppressants, corticosteroids, antibiotics, analgesics, vitamins, drugs for acid-related disorders, vaccines) were also tested using an indicator variable that changes from zero to one at the reported date of the event or exposure. Out of the quantitative variables, age was kept as quantitative; BMI was categorized according to WHO categories for underweight, normal weight, overweight, and obese (15.2–18.5, 18.5–25, 25–30, and 30–48.8 kg/m^2^, respectively); and smoking was divided into 3 categories (nonsmokers, intermediate smokers with 1–10 cigarettes smoked per day, and heavy smokers with 11–40 cigarettes per day) using the median of the number of cigarettes per day of the smokers to divide them into balanced groups. For taking into account multiple comparisons, we performed a false discovery rate (FDR)-based association analysis with a 5% FDR threshold for the bioclinical variables and the imputed HLA genotypes. Association of log-transformed CXCL12 protein levels with time to ADA was tested both with a linear model and with a 2-group qualitative variable dichotomized at median (672–2,488 pg/ml and 2,488–16,022 pg/ml), and the model with the best fit (lowest AIC) was retained. Association of log-transformed serum CXCL12 levels with the rs10508884 genotype was assessed through Welch's heteroscedastic F test with a global test (3 genotype groups) and 2 comparisons (recessive and dominant model). We also performed a linear regression on allele counts. The p-values for the 3 models (recessive, dominant, and additive) were Bonferroni-corrected.

The genome-wide association analysis for common genetic markers linked to the immunogenicity response was performed on the 457 patients whose DNA samples were available and who passed genotyping quality control checks.

We made no assumption about the underlying genetic model and considered an overall k-sample log-rank statistic (with k = 3 corresponding to the 3 genotypes: aa, aA, AA) for univariate survival analysis. This statistic is asymptotically distributed as a chi-square with 2 degrees of freedom [12]. In order to evaluate whether confounding due to population stratification exists, we computed the genomic control, which is defined as the median of the test statistics divided by its theoretical median under the null distribution. A ratio close to 1 indicates no stratification [13]. Because this study is an exploratory analysis, we performed an FDR-based genome-wide analysis at a less stringent 20% FDR threshold.

For the multivariate survival analyses, we used the Cox proportional hazards model [12]. For variables with less than 1% of missing data, we considered a simple mean imputation procedure in which missing values are replaced by the mean (continuous variables) or the mode (categorical variables).

#### Mediation analysis

**Fig 2:**
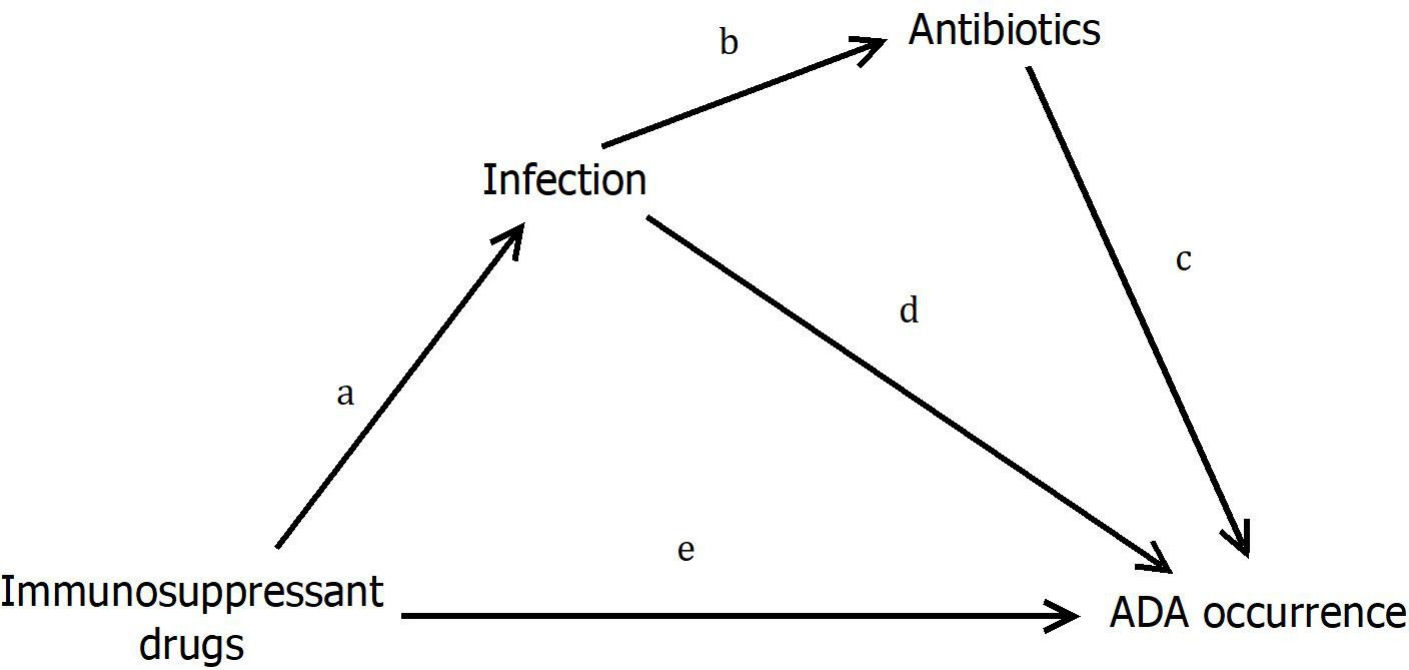
Diagram of the hypotheses tested through mediation analysis: Immunosuppressant drugs may induce infectious adverse events, which in turn may induce treatment with antibiotics for bacterial infections. Immunosuppressants have a direct effect on ADA occurrence (e) as well as indirect effects mediated through infection (a + d) and through antibiotics (a + b + c). ADA, antidrug antibody.

Based on the classical counterfactual framework and relying upon the pathway presented in Fig 2, we evaluated the direct and indirect effects of the immunosuppressants [14]. Because our main outcome is the time to ADA positivity, we considered for this analysis the Aalen additive hazard model [15], in which the rate of ADA occurrence was an additive function of the exposure to the immunosuppressants and the 2 mediators (infection and antibiotics). For this analysis, we used an indicator variable that changes from zero to one at the reported date of the event or exposure, and we adjusted for the variables that were previously found associated with ADAs. The relationships between immunosuppressants and infection and between infection and antibiotics were evaluated using a logistic regression model. Confidence intervals (CIs) for the direct effects (referred to as e, d, c in Fig 2) were obtained from the Aalen additive model, whereas CIs for the indirect effects (referred to as: a + d, a + b + c in Fig 2) were obtained by resampling.

## Results

### Demographic and clinical characteristics

In total, 609 patients were screened on entry into the study, out of whom 16 did not fulfill study criteria. After exclusion of second re-entry for another drug and baseline ADA test positive at entry, 560 patients were retained for the analysis. In total, 29 patients were recruited in Austria, 6 in Belgium, 50 in Czech Republic, 286 in France, 27 in Germany, 28 in Israel, 9 in Italy, 65 in the Netherlands, 22 in Spain, 10 in Sweden, 9 in Switzerland, and 19 in the United Kingdom. Of these, 560 patients were eligible for the survival analysis on bioclinical factors, from whom 457 were considered for the genome-wide association analysis (Fig 1). The flowchart gives the details of the patients recruited and eligible for the survival analysis and for the genetic analysis in each cohort. Table 1 and S1 Table display the demographic and clinical characteristics, respectively, at baseline in BP-treated patients stratified by disease cohort.

**Table 1:** Demographics of the ABIRISK cohorts. **Abbreviations:** ABIRISK, Anti-Biopharmaceutical Immunization: prediction and analysis of clinical relevance to minimize the RISK; BMI, body mass index; IBD, inflammatory bowel disease; IQR, interquartile range; MS, multiple sclerosis; RA, rheumatoid arthritis; SD, standard deviation.

		IBD, N = 184	MS, N = 147	RA, N = 229
**Age, mean (SD)**		36.9 (13.7)	35.1 (9.7)	54.2 (13.7)
**Sex, N (%)**	Female	89 (48.4)	103 (70.1)	176 (76.9)
	Male	95 (51.6)	44 (29.9)	53 (23.1)
**Smoke, N (%) (cigarettes per day)**	0	125 (68.3)	106 (72.6)	169 (74.8)
	1–10	34 (18.6)	18 (12.3)	33 (14.6)
	11–40	24 (13.1)	22 (15.1)	24 (10.6)
**BMI, N (%)**	Underweight	17 (9.3)	5 (3.4)	6 (2.7)
	Normal	114 (62.3)	84 (57.9)	110 (49.3)
	Overweight	40 (21.9)	29 (20)	56 (25.1)
	Obese	12 (6.6)	27 (18.6)	51 (22.9)
**Country, N (%)**	Austria	0 (0)	29 (19.7)	0 (0)
	Belgium	6 (3.3)	0 (0)	0 (0)
	Czech Republic	0 (0)	50 (34)	0 (0)
	France	150 (81.5)	0 (0)	136 (59.4)
	Germany	0 (0)	27 (18.4)	0 (0)
	Israel	28 (15.2)	0 (0)	0 (0)
	Italy	0 (0)	0 (0)	9 (3.9)
	Netherlands	0 (0)	0 (0)	65 (28.4)
	Spain	0 (0)	22 (15)	0 (0)
	Sweden	0 (0)	10 (6.8)	0 (0)
	Switzerland	0 (0)	9 (6.1)	0 (0)
	United Kingdom	0 (0)	0 (0)	19 (8.3)
**Follow-up, days; median (IQR)**		338.0 (63.5)	358.0 (175.0)	357.5 (158.25)

Among the 560 patients, we had 229 patients with RA, 184 patients with IBD, and 147 patients with MS. For RA and IBD, 153 patients were treated with adalimumab, 101 with infliximab, 84 with etanercept, 31 with rituximab, and 44 with tocilizumab. For MS, 38 patients were treated with IFN-beta-1a IM, 68 with IFN-beta-1a SC, and 41 with IFN-beta-1b SC. The most common route of drug administration was SC (66%), followed by intravenous (27%) and IM (7%). It is worth noting that this latter route is only used by a single treatment (IFN-beta-1a IM). The mean age of patients was significantly lower (p < 0.001) in MS (35.1 years) and IBD (36.9 years) as compared with RA (54.2 years). The proportion of female was significantly higher (p < 0.001) for RA (76.9%) and MS (70.1%) than for IBD (48.4%). Overall, 72% of the patients were not smoking, 15% were light smokers (1 to 10 cigarettes per day), and 13% were heavy smokers (11 to 40 cigarettes per day), with no statistical differences across the diseases.

BMI was significantly lower (p < 0.001) for IBD (23.1 kg/m^2^) than for RA (26.0 kg/m^2^) and MS (25.2 kg/m^2^). The mean disease duration before entry into the study was significantly shorter for MS (mean = 0.85 years) as compared to IBD (mean = 3.69 years) and RA (mean = 3.17 years, p < 0.001). The proportion of patients vaccinated with seasonal influenza or other vaccines in the year before study entry were higher (p < 0.001) for RA patients (37%) than for IBD (15%) and MS (5%). A family history of the same autoimmune diseases obtained from the questionnaires was significantly more often reported (p < 0.001) for IBD (18%) and RA (18%) than for MS (5%).

Table 2 displays the distribution of BPs and occurrence of ADA according to diseases and BPs. BPs are nested within the disease because IFN-beta is only given to MS patients, anti-CD20 and anti-IL6R are given only to RA patients, and TNF inhibitors are not given to MS patients. Thus, not all combinations of categories are represented.

**Table 2:** ADA occurrence during 12 months, stratified by disease and by BP therapy. ADA, antidrug antibody; BP, biopharmaceutical product; IBD, inflammatory bowel disease; IFN, interferon; IL, interleukin; IM, intramuscular; MS, multiple sclerosis; RA, rheumatoid arthritis; SC, subcutaneous; TNF, tumor necrosis factor.

Total N (ADA positive N)		IBD	MS	RA
**TNF inhibitors**	etanercept			84 (3)
	infliximab	86 (13)		15 (3)
	adalimumab	98 (38)		55 (26)
**IFN-beta**	IFNb-1a IM		38 (0)	
	IFNb-1a SC		68 (11)	
	IFNb-1b SC		41 (26)	
**Anti-CD20**	rituximab			31 (16)
**Anti-IL6-R**	tocilizumab			44 (4)

### Association between bioclinical variables and ADA occurrence

**Fig 3:**
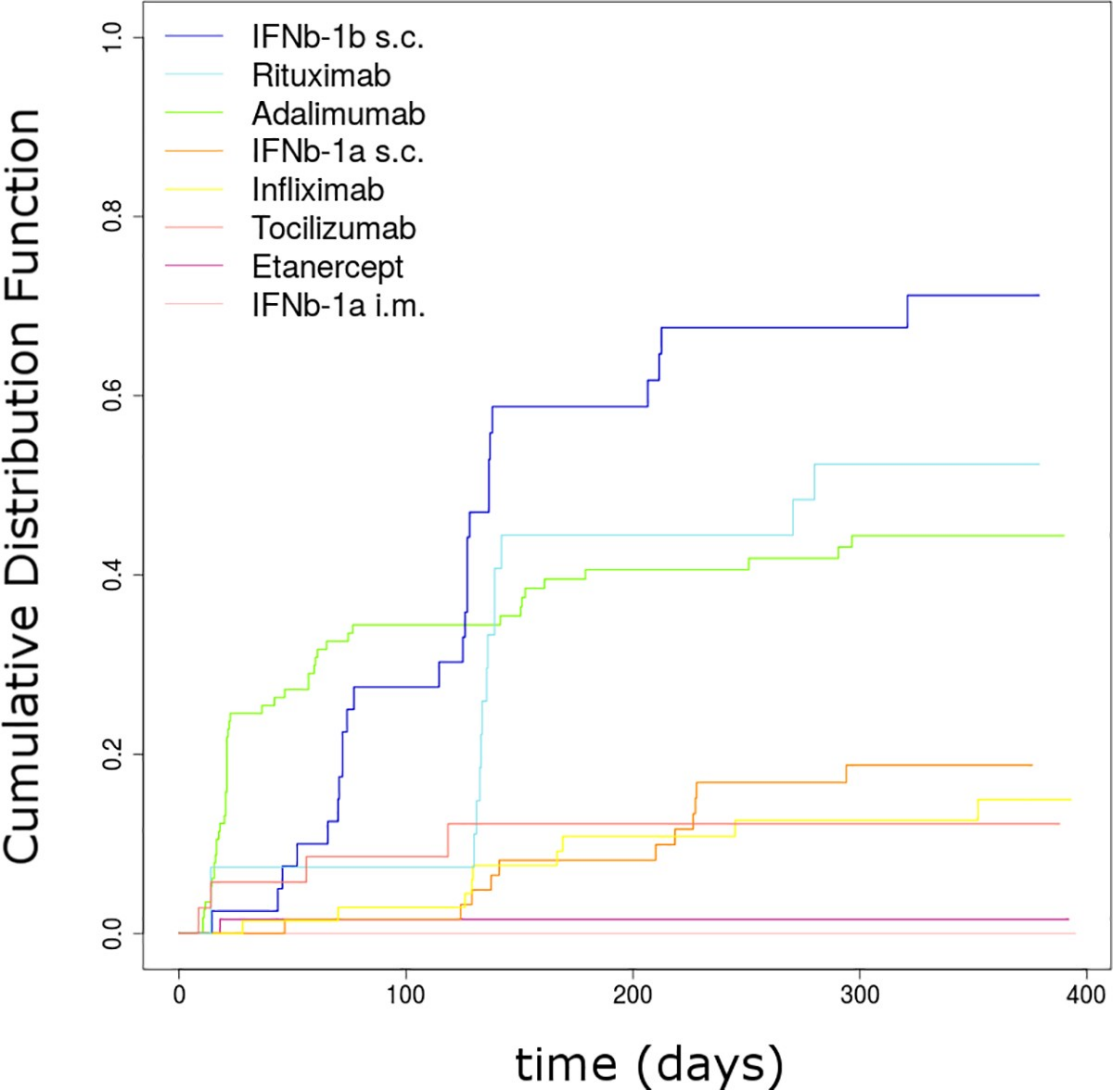
ADA occurrence by BP treatment. ADA, antidrug antibody; BP, biopharmaceutical product; IFNb, interferon beta; IM, intramuscular; SC, subcutaneous.

According to the univariate analyses, there was a statistically significant difference for immunogenicity across the drugs (p < 2 × 10^−16^). From Fig 3, we can broadly identify 3 groups. The first group, with very low ADA occurrence (less than 5%), is composed of etanercept and IFN-beta-1a IM. For this latter drug, we observed no ADAs among the 38 treated patients. The second group, with less than 20% of patients being positive for ADAs, is composed of tocilizumab, infliximab, and IFN-beta-1a SC. The third group, with more than 40% of patients being positive for ADAs, is composed of rituximab, adalimumab, and IFN-beta-1b SC, this latter drug exhibiting the highest occurrence of ADAs. When looking at the 4 groups of BPs based upon the mechanisms of action—that is to say, TNF inhibitors, IFN-beta, anti-CD20, and anti-IL6-R—there was a statistically significant difference, the latter group initiating the lowest rate of ADA production (p < 0.01).When looking at the results, it is worth noting that the variability of ADA rates between BPs from the same group was larger than the one across BPs from different mechanisms of action. Taking into account multiple comparisons, univariate analyses with stratification for disease showed that the clinical and demographic factors age, sex, BMI, recent vaccination, and familial history of autoimmune diseases were not significantly related to the time to ADA detection (Table 3). In contrast, tobacco smoking (nonsmokers or light smokers versus heavy smokers) was significant, with a higher risk of ADA for heavy smokers (hazard ratio [HR] = 1.756 [1.151–2.678], p < 0.01).

**Table 3:** Association of demographic and clinical variables to time to ADA through univariate Cox model stratified on disease. Selected factors for a 5% FDR threshold are indicated with an asterisk. **Abbreviations:** ADA, antidrug antibody; BMI, body mass index; CI, confidence interval; FDR, false discovery rate; HR, hazard ratio.

Variable	Statistic (Score)	HR (95% CI)	Unadjusted p-Value
**Age**	0.027	1.001 [0.989–1.014]	0.870
**Sex**	0.410	1.126 [0.7825–1.622]	0.522
**Smoke (nonsmoking or light smokers/heavy smokers)**	7.015	1.756 [1.151–2.678]	0.008*
**BMI (underweight or normal/overweight/obese)**	4.390	1.436 [1.022–2.016]	0.036
**Family history of same disease**	3.456	1.508 [0.975–2.333]	0.063
**Past infections**	2.817	1.558 [0.924–2.626]	0.093
**Hypertension**	0.0001	0.997 [0.462–2.148]	0.993
**Metabolic disease**	0.629	0.748 [0.364–1.536]	0.427
**Neoplastic disease**	0.374	1.270 [0.590–2.733]	0.540
**Nervous system disease**	0.356	1.244 [0.606–2.553]	0.550
**Respiratory disease**	0.032	0.933 [0.435–1.998]	0.858
**Past immunosuppressants**	0.026	1.040 [0.649–1.666]	0.870
**Past corticosteroids**	0.420	0.871 [0.573–1.323]	0.517
**Vaccines last year**	0.544	1.175 [0.765–1.805]	0.461
**Infections (time-dependent)**	10.111	2.223 [1.343–3.681]	0.001*
**Analgesics (time-dependent)**	0.0002	0.996 [0.630–1.575]	0.987
**Antibiotics (time-dependent)**	19.445	0.228 [0.111–0.467]	1.03 × 10^−5^*
**Corticosteroids (time-dependent)**	2.896	0.732 [0.511–1.050]	0.089
**Immunosuppressants (time-dependent)**	15.474	0.446 [0.296–0.671]	8.36 × 10^−5^*
**Vitamins (time-dependent)**	0.479	1.175 [0.744–1.857]	0.489
**Drugs for acid-related disorders (time-dependent)**	2.955	0.539 [0.264–1.103]	0.086
**Vaccines (time-dependent)**	2.641	0.528 [0.242–1.154]	0.104

**Fig 4:**
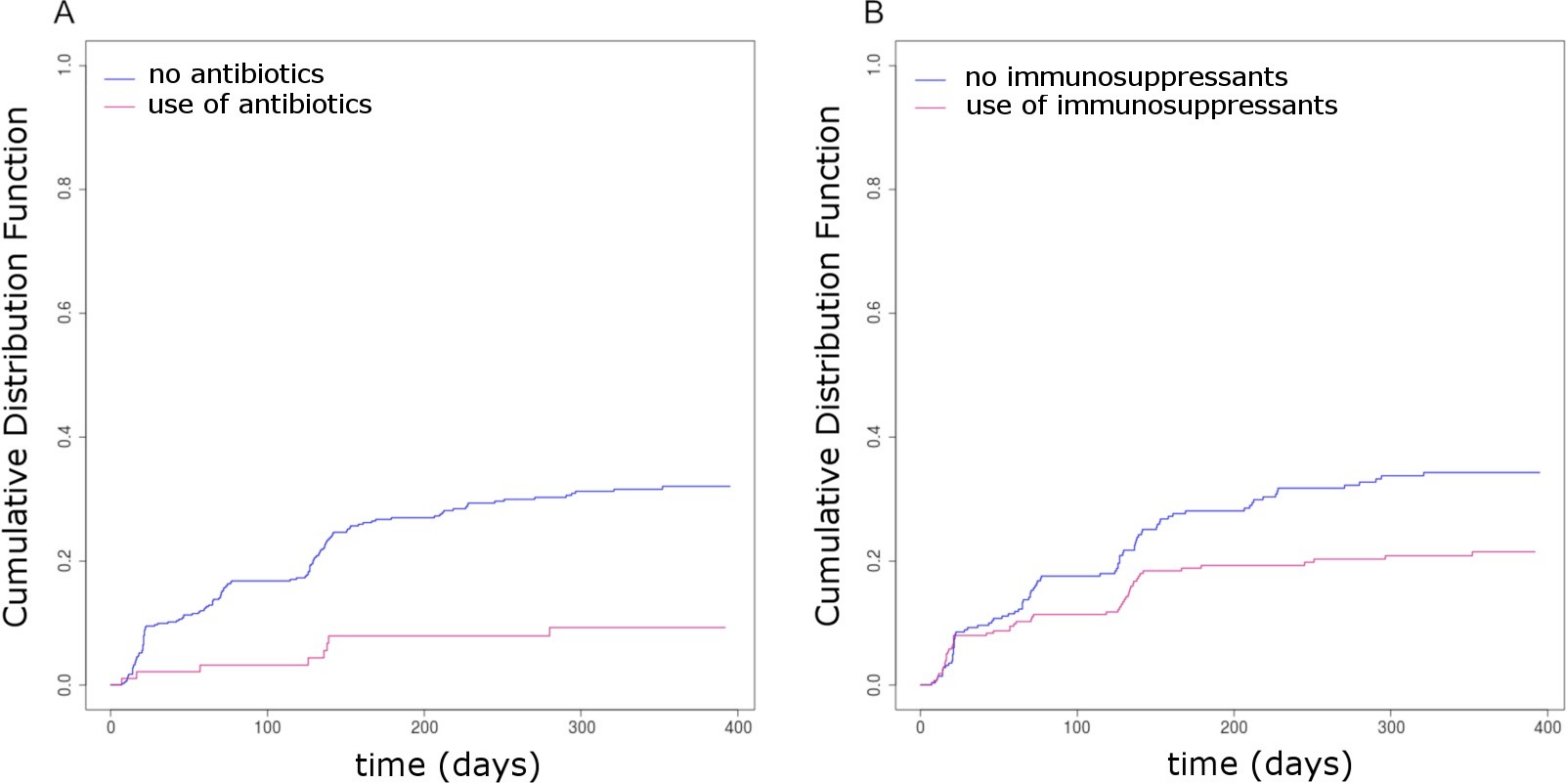
ADA occurrence according to the intake of (A) antibiotics or (B) immunosuppressants during the study. ADA, antidrug antibody.

When analyzing the potential effect of previous or concomitant medications taking into account multiple comparisons, univariate analyses showed that the time-dependent variables antibiotics (HR = 0.228 [0.111–0.467], p < 0.0001) (Fig 4A, S1 Fig) and immunosuppressive drugs (HR = 0.446 [0.296–0.671], p < 0.0001) (Fig 4B, S1 Fig) taken during the study were significantly associated with the time to ADA production. Patients receiving antibiotics and/or immunosuppressive drugs during the study have a lower risk of ADAs. In contrast, infections during the study were associated with an increased risk of ADAs (HR = 2.223 [1.343–3.681], p = 0.001). When including these variables in a multivariate survival model stratified on the disease, they were still significantly associated with the occurrence of ADAs (antibiotic adjusted hazard ratio [aHR] = 0.202 [0.098–0.418], p < 0.0001; immunosuppressive drugs aHR = 0.441 [0.294–0.663], p < 0.0001; infections aHR = 2.96 [1.8–4.897], p < 0.0001; heavy smokers versus low and nonsmokers aHR = 1.909 [1.246–2.923], p < 0.01).

When analyzing the relationship between these treatments and the disease, a higher proportion of patients with RA and IBDs were treated with immunosuppressive agents during the study (RA, 86% [81.0%–91.2%]; IBD, 53% [44.4%–61.5%]) as compared with MS (3% [0.1%–5.9%], p < 0.001), as expected from the usual therapeutic protocols. Moreover, a higher proportion of patients had received antibiotics during the study (p < 0.001) for RA (25% [19.3%–30.7%]) and IBDs (28% [21.2%–34.3%]) as compared with MS (7% [3.5%–11.1%]). There was a significantly positive relationship between the use of immunosuppressive drugs and antibiotics, with a higher percentage of patients with the 2 agents than expected by chance (p < 0.0005).

Knowing that immunosuppressant drugs may induce infectious adverse events and that antibiotics are usually given to the patients to treat bacterial infections, we considered that complex causal relationships between these 3 exposures and time to ADAs should exist that are not modeled by Cox regression. Therefore, we decided to perform a multiple mediation analysis according to the hypothesis described in Fig 2, adjusted for tobacco smoking. Results from the mediation analysis showed that the direct effects of immunosuppressants and antibiotics were associated with lower incidence of ADA such that they would decrease the number of cases by −4.97 × 10^−4^ [−8.53; −1.43] and −10.63 × 10^−4^ [−13.80; −7.41] persons per day per 10,000 individuals at risk, respectively. In contrast, the direct effect of infection was associated with higher incidence of ADA such that it would increase the number of cases by 11.17 × 10^−4^ [4.58; 17.80] persons per day per 10,000 individuals at risk. The indirect effect of immunosuppressants mediated by the infection led to a higher incidence of ADAs (1.34 × 10^−4^ [0.72; 1.96]).The indirect effect of immunosuppressants mediated by the infection and the antibiotics led to a lower incidence of ADA (−1.54 × 10^−4^ [−2.00; −1.08]). These results support the plausibility of the causal mechanisms hypothesized in Fig 2.

### Genetic variants associated with ADAs

**Fig 5:**
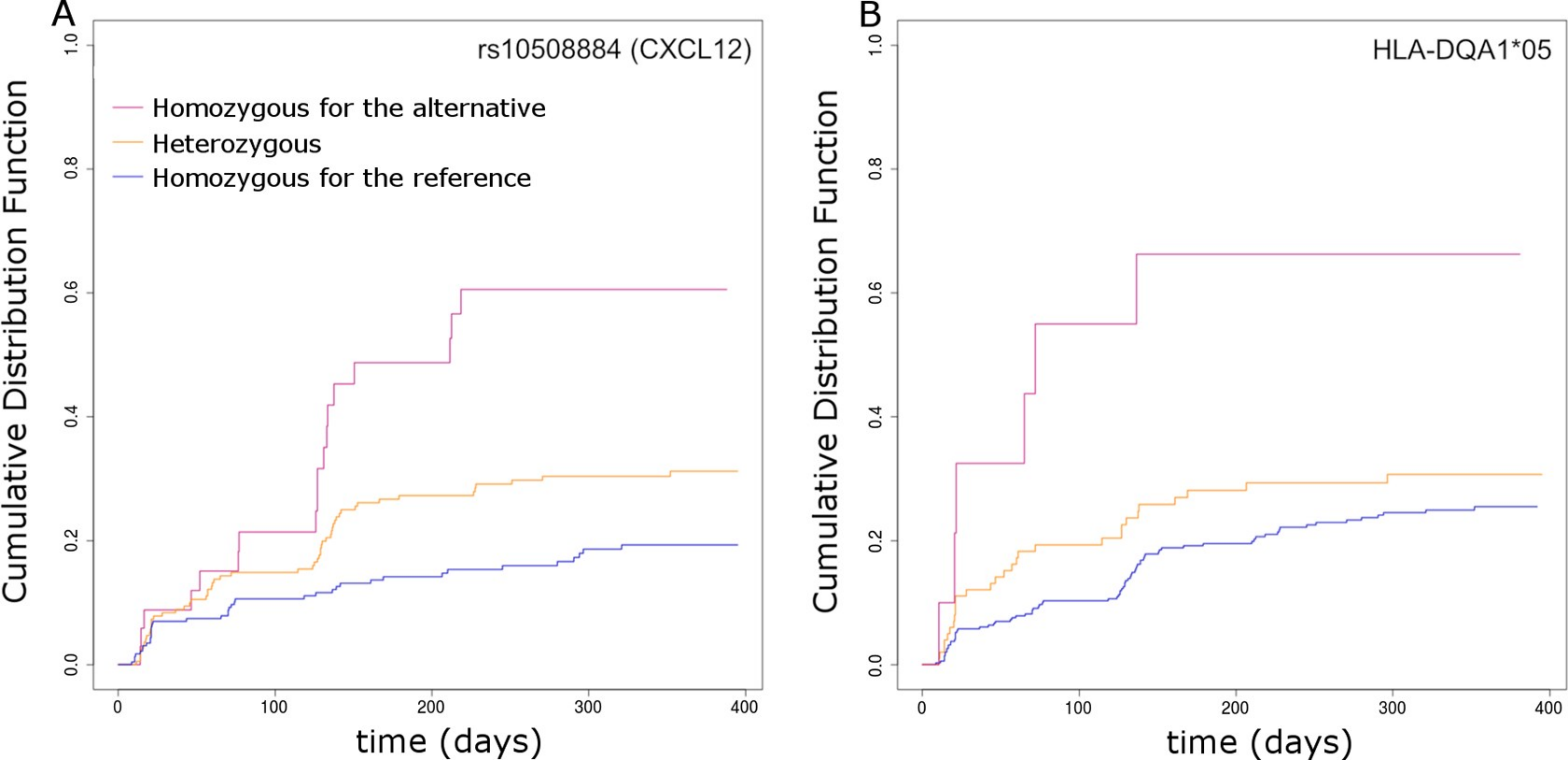
ADA occurrence according to (A) rs10508884 (*CXCL12*) or (B) HLA-DQA1*05. ADA, antidrug antibody; HLA, Human Leukocyte Antigen.

To identify genetic loci associated with ADAs, we performed an FDR-based genome-wide analysis (S2 Fig). Controlling the FDR at nominal level of 20%, we selected 6 associated signals: rs4879801 (*DNAI1*, Dynein intermediate chain 1, axonemal), rs1203638 (*PRDM2*, PR domain zinc finger protein 2), rs1626645 and rs894324 (*ASB7* region for the last 2, Ankyrin repeat and SOCS box protein 7), rs4879795 (*DNAI1*), and rs10508884 (*CXCL12*) (Table 4, Fig 5A). The value of the genomic control was close to one. There was no significant relationship between these SNPs and the disease (Bonferroni-corrected p-value = 1) or the population structure (using the first 10 principal components, Bonferroni-corrected p-value = 1). There was no relationship between these SNPs and the use of antibiotic (Bonferroni-corrected p-value = 0.18) and immunosuppressive drugs (Bonferroni-corrected p-value = 1) during the monitoring period. Two pairs of SNPs in the same gene located near each other were in complete (rs894324 and rs1626645) or near-complete (rs4879795 and rs487981) linkage disequilibrium.

**Table 4:** SNPs associated with time to ADA occurrence at 20% FDR using a Cox regression model stratified on the disease, without genetic model hypothesis. **Abbreviations:** ADA, antidrug antibody; ASB7, Ankyrin repeat and SOCS box protein 7; Chr, chromosome; CI, confidence interval; DNAI1, Dynein intermediate chain 1, axonemal; FDR, false discovery rate; HR, hazard ratio; MAF, Minor Allele Frequency; PRDM2, PR domain zinc finger protein 2; SNP, Single-Nucleotide Polymorphism.

SNP	MAF	Chr	Genomic Region	Test Statistic (Score)	Unadjusted p-Value	Genotype	N	HR	95% CI
rs4879801	0.34	9	DNAI1	28.99	5.06 × 10^−7^	[AA]	192	1	reference
						[Aa]	215	1.14	[0.74–1.74]
						[aa]	50	3.34	[2.02–5.5]
rs1203638	0.21	1	PRDM2	28.54	6.36 × 10^−7^	[AA]	283	1	reference
						[Aa]	156	0.77	[0.50–1.18]
						[aa]	18	4.03	[2.17–7.49]
rs1626645	0.20	15	ASB7	28.61	6.14 × 10^−7^	[AA]	290	1	reference
						[Aa]	151	1.15	[0.77–1.73]
						[aa]	16	4.92	[2.58–9.37]
rs894324	0.20	15	ASB7	28.61	6.14 × 10^−7^	[AA]	290	1	reference
						[Aa]	151	1.15	[0.77–1.73]
						[aa]	16	4.92	[2.58–9.37]
rs4879795	0.35	9	DNAI1	26.08	2.17 × 10^−6^	[AA]	194	1	reference
						[Aa]	209	1.17	[0.76–1.79]
						[aa]	54	3.16	[1.92–5.18]
rs10508884	0.29	10	CXCL12	25.11	3.52 × 10^−6^	[AA]	230	1	reference
						[Aa]	193	1.74	[1.16–2.63]
						[aa]	34	3.92	[2.22–6.89]

When analyzing imputed *HLA* genotypes and taking multiple testing into account, we found 4 HLA alleles associated to time to ADAs: DQB1*02, DRB1*11, DRB1*03, and DQA1*05 (S3 Table), out of which 3 pairs of alleles are in incomplete linkage disequilibrium: DQA1*05 and DRB1*03, DQA1*05 and DRB1*11, and DQB1*02 and DRB1*03 (S3 Fig). The DQA1*05 allele was associated with a higher risk of developing ADAs (1.81 [1.39–2.36], p < 1 × 10^−5^; Fig 5B).

Among the 6 selected SNPs, rs10508884 was situated in an intron of the *CXCL12* gene, coding for a chemokine known to have functions in affinity maturation of antibodies and in plasma cell survival and therefore a relevant candidate for inducing ADA development.

For the 457 individuals with available genetic information, we performed a multivariate Cox model stratified on the disease including rs10508884 (*CXCL12*), DQA1*05, immunosuppressants, infections, antibiotics during the study, and tobacco smoking as covariates. The associations with time to ADAs were still significant and consistent with what previously observed in univariate and mediation analyses (Table 5). After adjustment for these latter variables, none of the other 3 HLA alleles (DQB1*02, DRB1*11, and DRB1*03) were significantly associated with ADAs.

**Table 5:** Results of multivariate Cox regression of time to ADAs on infections, immunosuppressants, antibiotics, tobacco smoking, rs10508884 SNP, and HLA (DQA1*05) with stratification on the disease status. ADA, antidrug antibody; CI−, lower 95% confidence interval; CI+, upper 95% confidence interval; HLA, Human Leukocyte Antigen; HR, hazard ratio; SNP, Single-Nucleotide Polymorphism.

N = 457		**HR**	**CI− (0.95)**	**CI+ (0.95)**	**p-Value**
**Tobacco (heavy smokers)**	yes	2.150	1.319	3.503	0.002
**Infections**	yes	2.757	1.616	4.704	0.0002
**Immunosuppressants**	yes	0.408	0.253	0.657	0.0002
**Antibiotics**	yes	0.121	0.0437	0.333	4.5 × 10^−5^
**rs10508884**	[Aa]	1.901	1.254	2.883	0.0025
	[aa]	3.804	2.139	6.764	5.4 × 10^−6^
***HLA (DQA1*:*05)***	[Aa]	1.474	0.983	2.211	0.0605
	[aa]	3.900	1.923	5.976	2.4 × 10^−5^

### CXCL12 serum levels analysis

**Fig 6:**
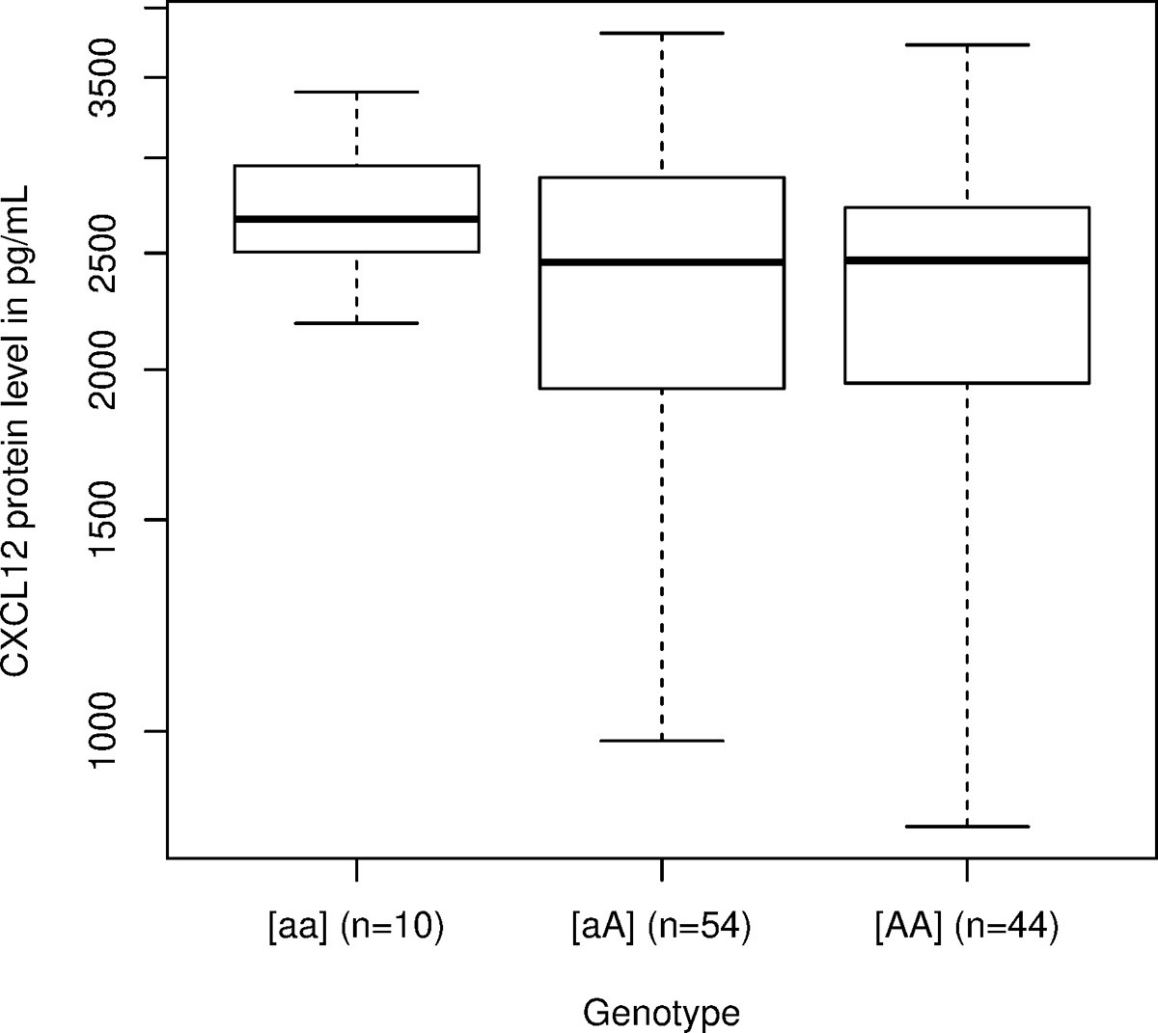
Boxplot of the CXCL12 serum levels according to genetic variant rs10508884 (*CXCL12*). The horizontal bars represent the median; whiskers span from the maximum to the minimum value.

An analysis of CXCL12 serum levels was performed on 108 adalimumab, infliximab, rituximab, or tocilizumab-treated patients suffering from RA. We tested for association between CXCL12 baseline serum levels (log-transformed values) and the genotype for the *CXCL12* variant rs10508884. We found that the concentrations of CXCL12 were significantly different between the 3 genotype groups (homozygotes of the minor allele, mean 2,693.374 [2,432.247–2,982.532] pg/ml; heterozygotes, mean 2,401.726 [2,187.21–2,637.279] pg/ml; homozygotes of the major allele, mean 2,218.492 [1,995.945–2,465.85]; p-value = 0.02). We found that the concentrations of CXCL12 were significantly higher (p-value = 0.014, Bonferroni-corrected p-value = 0.042) for the patients homozygous (mean: 2,693 pg/ml) for the minor allele than for the patients heterozygous or homozygous for the reference allele (mean: 2,317 pg/ml) at this locus (Fig 6). Neither the dominant model (unadjusted p-value = 0.14) nor the additive model (unadjusted p-value = 0.076) was significant. On the 108 analyzed patients when dichotomizing the CXCL12 serum levels at the median (2,488 pg/ml), we found that high concentrations of CXCL12 were associated with increased risk of ADA occurrence (HR = 2.59 [1.27–5.28], p < 0.01). When including CXCL12 serum levels in a multivariate Cox regression model, the relationship between CXCL12 and ADA risk was still significant (aHR = 2.329 [1.106–4.90]), as well as the relationship between ADA occurrence and antibiotics (aHR = 0.243 [0.072–0.819]) and infections (aHR = 2.867 [1.281–6.415]), whereas relationships with tobacco (aHR = 1.583 [0.532–4.710], p = 0.41) and immunosuppressants (aHR = 0.889 [0.387–2.040], p = 0.78) did not reach statistical significance.

## Discussion

The similar dynamic of ADA production across different BPs, the high immunogenicity of BPs when given to patients with autoimmune disease, and the immune hyperresponsiveness characteristic of these diseases suggest that ADA development may share common immunogenetic pathways. In the search for such common pathways, we conducted bioclinical survival analyses for immunogenicity development across MS, RA, Crohn's disease, and ulcerative colitis treated with various BPs. In this study, we were able to identify new clinical and genetic factors associated with ADA development (antibiotics, infections, a *CXCL12* genetic variant and CXCL12 serum protein levels) and to confirm ADA-associated factors identified in previous studies (tobacco smoking, immunosuppressants, and the HLA DQA1*05 allele).

In addition to increasing power gains, focusing on common patient-related factors associated with immunogenicity sheds new light on some common clinical and genetic factors that may be of interest for the coming clinical trials conducted with biotherapies.

Our study was conducted in the first 12 months of BP therapy, during which the occurrence of ADA in patients is the most frequent [16–18]. The use of highly sensitive ADA assays validated and performed within ABIRISK central laboratories [7–9] improved the power of the analysis by increasing the number of events detected and by allowing an earlier detection of ADA occurrence.

Informative censoring was not a concern because during the study, there were only 5 censored patients who switched to another drug, and 4 of them had negative ADA tests for at least 3 months after switch.

In our study, the analysis of clinical data identified concomitant immunosuppressant and antibiotic intake during BP therapy as factors associated with lower risk of ADA development. If the effect of immunosuppressants was well known from previous studies on immunogenicity of adalimumab and infliximab in RA and IBDs [19,20], the relationship between antibiotics and immunogenicity is a new finding of the ABIRISK study, to the best of our knowledge. Although immunosuppressant and antibiotic therapies were correlated, their effects were additive.

Moreover, the antibiotics were associated with an apparently lower risk of occurrence of ADAs than the immunosuppressants. Our findings suggest that part of the relationship between immunosuppressants and ADA occurrence could be mediated by an indirect effect through antibiotic intake due to increased risk of infectious adverse events, shedding light on composite mechanisms of immunosuppression which had not been appreciated before. The main immunosuppressants prescribed in the cohort were methotrexate (MTX), azathioprine, and leflunomide. A mechanism of MTX-induced tolerization was recently proposed by ABIRISK collaborators, who demonstrated in BAFF (B-cell activated factor) transgenic mice that high levels of BAFF were necessary for MTX-induced tolerization and confirmed it in the anti-TNF-treated patients of the ABIRISK prospective study [21]. BAFF induced an increase in the CD73 enzyme on the B cell surface, which catalyzes conversion of extracellular AMP (Adenosine MonoPhosphate) into the immunosuppressant adenosine. By inducing release of AMP from B cells, in the presence of CD73 MTX causes an increase in adenosine production and differentiation of regulatory B cells, which protect from ADA development.

A negative association of antibiotics with ADA development is a novel, to our knowledge, finding elucidated by this study; however, it is not the first report of an immunosuppressive effect of antibiotics. The efficacy of unadjuvanted viral vaccines such as inactivated influenza vaccine and polio vaccine and of whole-cell pertussis vaccine was decreased in antibiotic-treated mice and humans [22–24]; moreover, the efficacy of Immune Checkpoint Inhibitor (ICI) therapy against epithelial tumors in patients and in mice tumor models is altered by antibiotics use [25]. In 2 of these reports, the immunosuppressant effect of antibiotics was directly linked to changes in the gut microbiome; for the viral vaccines, it was mediated by absence of flagellin-carrying bacteria, which stimulate plasma cell differentiation and antibody secretion by binding to TLR5 (Toll-Like Receptor 5) on plasma cells and on lymph node macrophages. For ICIs, it was mediated by absence of the species *Akkermansia muciniphila*, inducing infiltration of IL12-secreting CD4+ T cells in ICI-treated tumors. The effect of antibiotics on ADA occurrence could also be mediated by an altered gut microbiome. What is more difficult to explain, yet is an emerging theme of gut microbiome studies, is how bacteria in the gut may influence an immune response elsewhere in the body. Few studies have directly addressed the question: one systematic investigation of host–microbiome interaction through monocolonization of germ-free mice with 53 different bacterial strains found that 88% of the gut colonizing species could be recovered in the mesenteric lymph nodes, and 47% could also be found in SC lymph nodes and spleen [26]. Furthermore, numbers of F4/80+ mononuclear phagocytes and of RAR-related orphan receptor gamma (RORγ)+Helios− regulatory T cells in the gut correlated with their frequencies in SC lymph nodes and spleen, suggesting a migration of immune cell populations from the gut to other lymphoid organs. A direct proof of this migration pattern is still lacking. Recently, blood-borne microbial metabolites such as short-chain fatty acids were shown to promote CD8+ memory T cell development [27]. Although it is an interesting finding, antibiotic treatment would certainly not be a good strategy to prevent ADA occurrence because of other risks carried by these therapies, such as development of resistant bacterial strains, whereas concomitant immunosuppressant treatment seems a more appropriate solution because it is already used in clinical practice for TNF inhibitor treatments and improves their efficacy [28,29]. There may, however, be alternative, safer, clinically useful ways to modulate the microbiome in lieu of antibiotic use.

The finding that infections during the study increase the risk of ADA occurrence supports the hypothesis that in addition to gut microbiome, pathogenic microbes could also exert an adjuvant effect. This suggests that non-antibiotic–treated infections, caused by bacteria or not, during BP therapy should be carefully monitored because of the increased risk of ADA occurrence.

In the present study, we found an increased risk of ADA development in heavy smokers, previously identified in natalizumab and IFN-beta–treated MS patients [30,31] and in infliximab-treated RA patients [32]. However, another study on 37 MS patients treated with IFN-beta did not find an association of cotinine, a smoke nicotine metabolite, with ADAs [33]. We previously found sex to be associated with immunogenicity in MS patients [34], but the sex at increased risk was females for natalizumab and males for IFN-beta, suggesting that BP-specific mechanisms of immunogenicity were involved. Heterogeneity might in part account for the absence of this association in the present study, together with the lower number of patients of the cohort and thereby lower power to identify factors with a small effect size.

The genome-wide analysis allowed us to identify 6 SNPs, among which one was in an intron of the *CXCL12* gene (rs10508884). We found that this genetic variant was associated with time to ADA occurrence, shedding light on central immunological mechanisms of antibody responses. Thus, we performed an additional study in which CXCL12 serum levels from RA patients were assessed and showed a significant relationship between CXCL12 levels and *CXCL12* genotypes. CXCL12 is a homeostatic chemokine involved in development, hematopoiesis, and angiogenesis [35]. CXCL12 exists in 6 distinct isoforms with a different ability to bind to glycosaminoglycans on the extracellular matrix [35] and thereby build chemokine gradients to attract cells expressing CXCR4, the CXCL12 receptor. CXCL12γ is the isoform with the highest affinity to heparan sulfate and has been shown to play a pivotal role in the germinal center reaction in secondary lymphoid organs: both *CXCR4*-deficient mice and *CXCL12*gagtm mice who lack the γ and the other heparan sulfate binding isoforms lose the light zone–dark zone polarity in the germinal centers, with impaired affinity maturation and inability to produce high-affinity antibodies as a consequence [36,37]. CXCL12γ is expressed by reticular cells in the dark zone, where it attracts proliferating centroblasts during the process of somatic hypermutation [36,37]. The spatial separation of the somatic hypermutation in the dark zone from the selection of high-affinity antibodies carried out in the light zone is essential for an efficient affinity maturation [36,37]. Therefore, it may explain that polymorphisms within the *CXCL12* gene are associated with time to ADA occurrence. Furthermore, CXCL12 is also involved in the migration of plasma cells to bone marrow, where it is produced by stromal cells. This migration is necessary for long-term survival of plasma cells, which need to occupy a bone marrow niche and are responsible for the constitutive secretion of antibodies in the circulation [38]. A *CXCL12* SNP (rs266087) was previously found to be associated with antibody responses to cholera vaccine [39], and the expression of plasma cell survival factors, including CXCL12, was increased in high-titer antibody responders to measles vaccine [40], highlighting the importance of this chemokine in antibody responses. A small CXCR4 antagonist, AMD3100, is currently used in combination with G-CSF (Granulocyte Colony-Stimulating Factor) as stem-cell mobilizing agent to treat non-Hodgkin’s lymphoma and multiple myeloma [35]. AMD3100 in combination with G-CSF, anti-CD20 and IL2/anti-IL2 mAb (monoclonal Antibody) complexes has been tested as a treatment to induce tolerance in a mouse model of hemophilia A with ADAs against Factor VIII, and it achieved a successful stable reduction of long-lived ADA-producing plasma cells and of ADA titers [41]. Thus, CXCL12 could be an interesting drug target for the treatment and prevention of ADAs.

Our analysis of imputed *HLA* loci from SNPs information allowed us to identify an association between HLA DQA1*05 and the occurrence of ADA, with a higher risk of ADA occurrence in individuals carrying the rare allele. Our finding is in accordance with the recent genome-wide association study across 1,240 Crohn’s disease patients enrolled in the PANTS (Personalizing anti-TNF Therapy in Crohn’s disease) cohort showing that HLA-DQA1*05 is significantly associated with an increased rate of ADAs in patients treated with infliximab and adalimumab [42].

Previous studies in IFN-beta–treated MS patients and in anti-TNF–treated IBD and RA patients have shown association of ADAs with *HLA-DRB1* alleles, with specific alleles for each disease and BP potentially suggesting different capacity to present BP-specific peptides to T cells [43,44]. It is unlikely that HLA-DQA1*05 has a particular ability to present peptides from BPs, which have different protein sequences and are unlikely to display common features; the effect of this allele is more probably due to strong linkage disequilibrium with an eQTL (expression Quantitative Trait Locus) controlling the expression of HLA class II or other immune-related genes in the *HLA* locus. The presence of such eQTLs has been recently demonstrated for the MS-associated HLA-DRB1*15:*01* allele: this allele is in linkage disequilibrium with a differentially methylated region and induces a lower methylation of the *HLA-DR* gene and consequently an increased expression of HLA-DR on monocytes [45]. This polymorphism and methylation have been shown to be causal in MS. Furthermore, a high density of eQTLs has been found in the regions proximal to *HLA-C* and *HLA-DR*, with effects on the expression of multiple genes of the *HLA* locus in 3 studied haplotypes [46], and the *HLA* alleles associated with different autoimmune diseases all correlate with HLA class II expression [47]. The influence of the HLA-DQA1*05 allele on expression of HLA class II warrants further investigation.

Despite these promising results, some limitations of our study should be mentioned. The genetic study that we conducted was an exploratory analysis and must be confirmed by replication in an independent cohort. The joint analysis of different diseases and BPs allowed us to identify common factors of immunogenicity, but it does not allow us to detect BP–covariate or disease–covariate qualitative interactions due to BP-specific or disease-specific immunogenicity mechanisms; a separate analysis for each disease and BP will be needed to address this question.

From a clinical perspective, the relevance and the clinical threshold titer level of the ADAs detected with each assay still remain to be established. Moreover, unlike quantitative methods that use reference standards, ADA methods are highly dependent on the drug-specific PC antibodies. The performance of the ADA method is not based on a single human reference ADA but on a surrogate PC such as polyclonal or monoclonal antibody raised in a nonhuman species [48]. The PCs are used to develop each ADA assay and for system suitability control of each individual method over time. The sensitivity and drug tolerance of each BP method is therefore expected to vary between different assays. This limits the possibility of quantitatively comparing the immunogenicity of different BPs [49,50]. Furthermore, except for the TNF inhibitors adalimumab and infliximab, which are prescribed for both RA and IBDs, all the other BPs analyzed were specific for one pathology, which makes it difficult to distinguish the contribution of the disease versus the BP to ADA development. Nevertheless, 3 groups of drugs could be broadly defined: very low immunogenic BPs (IFN-beta-1a IM and etanercept), intermediate/medium immunogenic BPs (tocilizumab, IFN-beta-1a SC, infliximab), and high immunogenic BPs (adalimumab, IFN-beta-1b SC, rituximab). Notably, with the newly developed MSD assay, adalimumab gave a higher incidence of ADAs than infliximab in both RA and IBDs, in contrast to what has been described in previous reports from other research teams [51,52] and from an ABIRISK retrospective study [32]. Most of the previous studies have used ELISA BAb assays or radio-immuno assays and might have lacked sensitivity and/or tolerance to detect low titers of ADAs in the presence of circulating BPs because of the formation of BP–ADA immune complexes, an issue that has been solved by using a more drug-tolerant MSD assay in the present prospective study [53]. A pharmacokinetic measure of drug level for the TNF inhibitors and tocilizumab has been performed, but it will be the object of a separate analysis because for IFN-beta, the half-life is too short to allow a pharmacokinetic measurement, and a comparison of all the drugs included in this joint study was not possible. Tocilizumab and rituximab also resulted in a higher ADA incidence in the ABIRISK prospective study than in previous reports, likely because of the sensitivity and drug tolerance of the MSD assay, which has not been used before for detecting ADAs against these BPs in RA [54–57]. Concerning the other biologicals analyzed in this study, the broad immunogenicity level was comparable to what has been previously reported [58,59].

In conclusion, through the joint analysis of BP immunogenicity in 4 autoimmune diseases treated with different BPs, we have identified clinical (heavy smoking, concomitant immunosuppressants, antibiotics, and infections) and genetic (a *CXCL12* variant, HLA-DQA1*05 allele) risk factors associated with time to ADA occurrence pointing to more general mechanisms of immunogenicity. These results may help physicians in the management of patients receiving biotherapies.

## Supporting information

S1 Checklist STROBE checklist. STROBE, Strengthening the Reporting of Observational Studies in Epidemiology.
(PDF)

S1 Text Statistical analysis plan of the ABIRISK prospective study. ABIRISK, Anti-Biopharmaceutical Immunization: prediction and analysis of clinical relevance to minimize the RISK.
(PDF)

S1 Table Clinical characteristics at baseline in BP-treated patients stratified by disease cohort. ACPA, anti-citrullinated peptide antibodies; BP, biopharmaceutical product; CIS, Clinically Isolated Syndrome; DAS28, disease activity score 28; EDSS, expanded disability status scale; IBD, inflammatory bowel disease; IQR, interquartile range; MS, multiple sclerosis; RA, rheumatoid arthritis; RF, rheumatoid factor; RRMS, relapsing remitting multiple sclerosis.
(PDF)

S2 Table Missing data and MedDRA and WHODrug codes for the demographics and clinical variables. MedDRA, medical dictionary for regulatory activities; MS, multiple sclerosis; NA, nonapplicable; RA, rheumatoid arthritis; UC, ulcerative colitis; WHODrug, World Health Organization drug dictionary.
(DOCX)

S3 Table Results of univariate analyses on HLA alleles. df, degrees of freedom; FDR, false discovery rate; HLA, Human Leukocyte Antigen.
(XLSX)

S1 Fig ADA occurrence per disease (IBD, MS, RA) according to the intake of antibiotics or immunosuppressants during the study. ADA, antidrug antibody; IBD, inflammatory bowel disease; MS, multiple sclerosis; RA, rheumatoid arthritis.
(TIF)

S2 Fig GWAS results (Manhattan plot). Genomic coordinates on 23 chromosomes are displayed on the x-axis, and the negative logarithm of the association p-value with ADA occurrence for each SNP is displayed on the y-axis. The blue horizontal dotted line represents the threshold of significance with a 20% FDR, and the red dots are the SNPs above the threshold. ADA, antidrug antibody; FDR, false discovery rate; GWAS, genome-wide association study; SNP, Single-Nucleotide Polymorphism.
(PDF)

S3 Fig Linkage disequilibrium plot of analyzed HLA alleles. Each square represents the LD measured by r^2^ between a pair of HLA alleles, listed on top of the plot according to their topographical order of appearance on chromosome 6. The color scale goes from white, corresponding to r^2^ = 0, to red, corresponding to r^2^ = 1. HLA, Human Leukocyte Antigen; LD, linkage disequilibrium.
(TIF)

## Abbreviations

ABIRISK: Anti-Biopharmaceutical Immunization: prediction and analysis of clinical relevance to minimize the RISK
ADA: antidrug antibody
aHR: adjusted hazard ratio
AMP: Adenosine MonoPhosphate
ASB7: Ankyrin repeat and SOCS box protein 7
ATC2: Anatomic Therapeutic Chemical level 2
BAb: binding antibody
BAFF: B-cell–activating factor
BMI: body mass index
BP: biopharmaceutical product
CD20: Cluster of Differentiation 20
CI: confidence interval
DNA1: Dynein intermediate chain 1, axonemal (gene)
eCRF: electronic Case Report Form
ELISA: enzyme-linked immunosorbent assay
eQTL: expression Quantitative Trait Loci
FDR: false discovery rate
G-CSF: Granulocyte Colony-Stimulating Factor
HLA: Human Leukocyte Antigen
HLGT: High-Level Group Term
HR: hazard ratio
IBD: inflammatory bowel disease
ICI: Immune Checkpoint Inhibitor
IFN: interferon
IL: interleukin
IM: intramuscular(ly)
LLT: Lowest-Level Term
mAb: monoclonal Antibody
MAF: Minor Allele Frequency
MPA: Medical Product Agency
MS: multiple sclerosis
MSD: MesoScale Discovery
MTX: methotrexate
NAb: Neutralizing Antibody
PANTS: Personalizing anti-TNF Therapy in Crohn’s disease
PC: positive control
PRDM2: PR domain zinc finger protein 2
PT: Preferred Term
RA: rheumatoid arthritis
RORγ: RAR-related orphan receptor gamma
SC: subcutaneous(ly)
SD: standard deviation
SNP: Single-Nucleotide Polymorphism
SOC: System Organ Class
STROBE: Strengthening the Reporting of Observational Studies in Epidemiology
TLR5: Toll-Like Receptor 5
TNF: tumor necrosis factor
