# Supplementary material for: Clinicogenomic factors of biotherapy immunogenicity in autoimmune disease: A prospective multicohort study of the ABIRISK consortium
Source: PLoS Med. 2020 Oct 30;17(10):e1003348. doi: 10.1371/journal.pmed.1003348 (PMC7598520; doi:10.1371/journal.pmed.1003348)
Supplement: S1 Checklist — STROBE, Strengthening the Reporting of Observational Studies in Epidemiology. (PDF) [file pmed.1003348.s001.pdf]

S1 Checklist

STROBE Statement—Checklist of items that should be included in reports of ***cohort studies***

	Item No	Recommendation	Section
**Title and abstract**	1	(*a*) Indicate the study's design with a commonly used term in the title or the abstract	YES Title
		(*b*) Provide in the abstract an informative and balanced summary of what was done and what was found	YES Abstract
**Introduction**			
Background/rationale	2	Explain the scientific background and rationale for the investigation being reported	YES Intro, paragraphs 1 & 2
Objectives	3	State specific objectives, including any prespecified hypotheses	YES Intro, paragraphs 3 & 4
**Methods**			
Study design	4	Present key elements of study design early in the paper	YES M&M, Study design and patient characteristics, paragraph 1
Setting	5	Describe the setting, locations, and relevant dates, including periods of recruitment, exposure, follow-up, and data collection	YES M&M, Study design and patient characteristics
Participants	6	(*a*) Give the eligibility criteria, and the sources and methods of selection of participants. Describe methods of follow-up	YES M&M, Study design and patient characteristics, paragraphs 2-5
		(*b*) For matched studies, give matching criteria and number of exposed and unexposed	Not applicable
Variables	7	Clearly define all outcomes, exposures, predictors, potential confounders, and effect modifiers. Give diagnostic criteria, if applicable	YES M&M, Study design and patient characteristics, paragraphs 3-5 Time-to-event outcomes, Survival analysis
Data sources/ measurement	8*	For each variable of interest, give sources of data and details of methods of assessment (measurement). Describe comparability of assessment methods if there is more than one group	YES M&M, Study design and patient characteristics, paragraphs 2 & 6, Anti-drug antibody assays, CXCL12 laboratory test, DNA extraction and genotyping
Bias	9	Describe any efforts to address potential sources of bias	YES M&M, Survival analysis, paragraph 3
Study size	10	Explain how the study size was arrived at	Not applicable (real-life prospective study)
Quantitative variables	11	Explain how quantitative variables were handled in the analyses. If applicable, describe which groupings were chosen and	YES M&M, Survival analysis, paragraph 1

		why	
Statistical methods	12	(*a*) Describe all statistical methods, including those used to control for confounding	YES<br>M&M, Survival analysis, Mediation analysis
		(*b*) Describe any methods used to examine subgroups and interactions	Not applicable
		(*c*) Explain how missing data were addressed	YES<br>M&M, Survival analysis, paragraph 4
		(*d*) If applicable, explain how loss to follow-up was addressed	YES<br>M&M, Time-to-event outcomes
		(*e*) Describe any sensitivity analyses	Not applicable
**Results**			
Participants	13*	(a) Report numbers of individuals at each stage of study—eg numbers potentially eligible, examined for eligibility, confirmed eligible, included in the study, completing follow-up, and analysed	YES<br>M&M<br>Flowchart Fig 1
		(b) Give reasons for non-participation at each stage	YES<br>M&M<br>Flowchart Fig 1
		(c) Consider use of a flow diagram	YES<br>M&M<br>Flowchart Fig 1
Descriptive data	14*	(a) Give characteristics of study participants (eg demographic, clinical, social) and information on exposures and potential confounders	YES<br>Results, Demographic and clinical characteristics, paragraphs 1-3<br>Table1<br>S1 Table
		(b) Indicate number of participants with missing data for each variable of interest	YES<br>Flowchart Fig 1<br>S2 Table
		(c) Summarise follow-up time (eg, average and total amount)	YES<br>Table 1
Outcome data	15*	Report numbers of outcome events or summary measures over time	YES<br>Results, Demographic and clinical characteristics, paragraph 4<br>Table 2
Main results	16	(*a*) Give unadjusted estimates and, if applicable, confounder-adjusted estimates and their precision (eg, 95% confidence interval). Make clear which confounders were adjusted for and why they were included	YES<br>Results, Association between bio-clinical variables and ADA occurrence, Genetic variants associated with ADAs, CXCL12 serum level analysis<br>Table 3<br>Table 4<br>Table 5

		(*b*) Report category boundaries when continuous variables were categorized	YES<br>M&M, Survival analysis, paragraph 1
		(*c*) If relevant, consider translating estimates of relative risk into absolute risk for a meaningful time period	YES<br>Results, Association between bio-clinical variables and ADA occurrence, paragraph 4 (mediation analysis)
Other analyses	17	Report other analyses done—eg analyses of subgroups and interactions, and sensitivity analyses	YES<br>Results, CXCL12 serum level analysis
**Discussion**			
Key results	18	Summarise key results with reference to study objectives	YES<br>Disc., paragraph 1
Limitations	19	Discuss limitations of the study, taking into account sources of potential bias or imprecision. Discuss both direction and magnitude of any potential bias	YES<br>Disc., paragraphs 12-13
Interpretation	20	Give a cautious overall interpretation of results considering objectives, limitations, multiplicity of analyses, results from similar studies, and other relevant evidence	YES<br>Disc., paragraphs 5-11
Generalisability	21	Discuss the generalisability (external validity) of the study results	YES<br>Disc., paragraph 12
**Other information**			
Funding	22	Give the source of funding and the role of the funders for the present study and, if applicable, for the original study on which the present article is based	YES<br>Funding statement

*Give information separately for exposed and unexposed groups.

Note: An Explanation and Elaboration article discusses each checklist item and gives methodological background and published examples of transparent reporting. The STROBE checklist is best used in conjunction with this article (freely available on the Web sites of PLoS Medicine at http://www.plosmedicine.org/, Annals of Internal Medicine at http://www.annals.org/, and Epidemiology at http://www.epidem.com/). Information on the STROBE Initiative is available at http://www.strobe-statement.org.
