# Supplementary material for: Clinicogenomic factors of biotherapy immunogenicity in autoimmune disease: A prospective multicohort study of the ABIRISK consortium
Source: PLoS Med. 2020 Oct 30;17(10):e1003348. doi: 10.1371/journal.pmed.1003348 (PMC7598520; doi:10.1371/journal.pmed.1003348)
Supplement: S1 Text — ABIRISK, Anti-Biopharmaceutical Immunization: prediction and analysis of clinical relevance to minimize the RISK. (PDF) [file pmed.1003348.s002.pdf]

S1 Text. Statistical analysis plan of the ABIRISK prospective study.

Deviations from the initial analysis plan

The statistical analysis plan was written in June 2017 before starting the analyses. Apart from minor methodological modifications (e.g. use of standard right censored survival analysis instead of interval-censored) most of the described analyses have been performed, but due to the large amount of data and results and to the further methodological development needed on the random survival forests we decided to split this work in two publications:

1) A clinical article on the association analysis that we are submitting to PLOS Medicine

2) A methodological article on the random survival forest prediction analysis that will be the object of another article

The CXCL12 protein assay in patient sera had not been planned from the beginning, it was decided after analyzing the GWAS results as CXCL12 seemed the most promising candidate for validation due to its already known immunological function.

**Title:**	**Prediction of ADA development across MS, RA and IBD patients treated with biopharmaceutical products**
**Effective Date:**	June 2017

WP4

Authors: Hässler Signe, Bachelet Delphine, Mbogning Cyprien and Broët Philippe

1. INTRODUCTION

- **Background**

The ABIRISK (Anti-biopharmaceutical Immunization: Prediction and analysis of clinical relevance to minimize the risk) consortium, within the IMI (Innovative Medicines Initiative), is a Public Private Partnership between pharmaceutical companies, small and medium-sized enterprises, academic institutions and clinical centres. The ABIRISK consortium aims are to better analyse and predict the phenomenon of immunogenicity in order to reduce its occurrence.

The number of biological/biotechnology-derived proteins used as therapeutic agents (called Biopharmaceuticals) is steadily increasing. Biopharmaceuticals (BPs) may induce an unwanted immune response in treated patients. Immunogenicity related to BPs therapy refers to a specific anti-drug antibody (ADA) response. Such immunogenicity may represent a major factor impairing the efficacy of BP due to BP neutralization and also to hypersensitivity reactions that are IgE or non-IgE-mediated. Production of ADA represents the final stage of a complex immune process involving antigen presentation followed by activation of both adaptive and regulatory cellular immune responses. Therefore, in order to minimize the risk of ADA induction and improve sustainable BP efficacy, ABIRISK project is focusing its efforts on understanding mechanisms by which BPs drive immune cell activation and ADA production mainly in four diseases: hemophilia A (HA), multiple sclerosis (MS), rheumatoid arthritis (RA), and inflammatory bowel diseases (IBD).

- **Objective**

In this context, a multicentric prospective study has been launched which is multi-drugs, multi-diseases (IBD, MS, RA), multicentric and focuses on the occurrence of ADAs.

Even though, MS, RA and IBD have very different phenotypic manifestations, there are some arguments suggesting that they share some common bio-clinical and genetic factors for ADA occurrence.

Based upon this hypothesis, we consider a joint time-to-event analysis where the main endpoint is the time to first ADA positivity.

2. STRATEGY

To predict the occurrence of ADAs in the first year of BP's treatments across three autoimmune disorders (MS, RA and IBD) and to select bio-clinical markers associated with the occurrence of ADAs.

For this joint analysis, we have obtained high-dimensional genomic data (constitutional DNA) from high-throughput genomic technologies (microarrays). Thus, for predicting ADA occurrence, we have to cope with high-order gene-gene interactions that play a major role within biological pathways. These interactions are frequently ignored more for reasons related with technical issues than biology considerations. Indeed, the extremely large

number of potential interactions prevent from being specified in advance and incorporated in classical regression models and any exhaustive search of high order interactions between all loci is computationally impractical.

In this context, tree-based recursive partitioning methods provide well-suited and powerful alternatives. This nonparametric methodology partitions recursively the predictor space into disjoint sub-regions (so-called terminal nodes or leaves) that are near homogeneous according to the outcome of interest. This framework is particularly well-suited to detect relevant interactions and produce prediction in high-dimensional data and it has been extended to survival data (termed as survival trees). The key component for survival trees is the splitting criterion which relies either on minimizing the within-node homogeneity or maximizing the between-node heterogeneity. However, the well-known instability of tree-based structures advocates for the use of so-called survival ensemble methods where the trees are the base learners.

Thus, for this analysis, we will consider the random survival forest framework.

Moreover, there is a special issue that directly affects the predictive accuracy and relates to the fact that we have in this study a mixed population of immune-reactive and immune-tolerant patients where the immune-tolerant ones will not experience ADA in the long term follow-up. This problem requires to consider a specific approach.

In practice, for this Abirisk project, we have developed a new and specific procedure that relies upon a bagging improper survival tree. This latter is well-suited for solving the issues related with high-order gene-gene interactions and mixed populations.

This procedure will be considered for the joint analysis

3. METHODOLOGY

Analysis Population

A population eligible for inclusion in the analysis will be selected. The eligible patients must meet all the inclusion criteria and none of the exclusion criteria of the clinical protocol of one of the ABIRISK prospective studies (see clinical protocols ABI-MS-P01, ABI-RA-P01, ABI-IBD-P01).

A patient will be excluded for the final analysis if he/she drops out immediately after the inclusion or the beginning of the treatment. In other words, we require that a patient has performed at least one visit after the inclusion and after the start of the BP treatment.

An additional criterion for this joint analysis is that the patient must have given informed consent for genetic analysis and he must have genotyping data available.

Summary of eligibility criteria:

- Patient with diagnosed CIS, RRMS, RA, Crohn's disease or ulcerative colitis who has been prescribed a BP treatment

- At least one dose of BP treatment and at least 1 ADA test result in period post first treatment date
- BP naïve to the selected BP prior to current study
- Age at first treatment more than 18 years
- Genotyping data available

Study Design

This is a multicentric prospective study across three different autoimmune disorders (MS, RA and IBD). Patient cohorts are recruited in several hospitals in European countries. The studies were approved by ethical committees in each of the countries involved.

Patients with MS, RA or IBD who had been prescribed a BP by a physician independently of the study were followed for 12 months from the start of the therapy, during which 7 to 12 visits were performed (specific to each disease study protocol), clinical and epidemiological data were recorded, DNA samples and serum samples were collected for genetic analyses and ADA testing respectively. The study period was 2013-2017. The BP treatments selected for the study were IFNβ for MS, infliximab and its biosimilars, adalimumab, etanercept, rituximab and tocilizumab for RA, infliximab and its biosimilars and adalimumab for IBD.

All the data from the different sites were gathered in a unique database (TranSMART) hosted by the eTRIKS consortium following standardized data loading procedures according to CDISC terminology.

Primary Outcome

1. Anti-drug antibodies assay protocols

Anti-drug antibodies were detected by specific validated assays for each BP and analyzed in central ABIRISK laboratories.

For IFNβ binding antibodies (BAbs) were tested with an ELISA at the University of Düsseldorf, Department of Neurology, Düsseldorf, Germany, and neutralizing antibodies (NAbs) with a functional luciferase assay in Region Hovedstaden laboratory, Copenhagen. When NAbs were positive a titration was performed.
For etanercept, infliximab (and its biosimilars) BAbs were tested with specific bridge-ELISAs (LISA-TRACKER Theradiag) at the clinical immunology laboratory of the Kremlin-Bicêtre hospital. For adalimumab BAbs were tested with a Meso Scale Discovery (MSD) assay at the clinical immunology laboratory of the Kremlin Bicêtre hospital. For rituximab BAbs were tested with an MSD assay at the clinical immunology laboratory of GlaxoSmithKline Research and Development, Upper Merion, PA, USA. For tocilizumab BAbs were tested with an MSD assay at the Eurodiagnostica AB laboratory in Malmö, Sweden.

2. Positive ADA definition

Since the assays are specific to each BP, and for some BPs more than one type of assay is run, the ADA positivity is defined by specific criteria for each BP in order to achieve a quite similar biologically meaningful definition for all the BPs. Thus, a serum sample is defined as ADA positive as follows:

- A serum sample is defined as IFNβ ADA positive if it is either BAb positive or NAb positive (with a NAb titer equal or higher than 320 TRU/ML), or positive for both criteria.

- A serum sample is defined as adalimumab ADA positive if it is positive for MSD assay.

- A serum sample is defined as infliximab (and its biosimilars) ADA positive if it is BAb positive.

- A serum sample is defined as etanercept ADA positive if it is BAb positive.

- A serum sample is defined as rituximab ADA positive if it is positive for MSD assay.

- A serum sample is defined as tocilizumab ADA positive if it is positive for MSD assay.

3. Outcome definition

The time-to-event (ADA positivity) is calculated from the date of first treatment to the time of first ADA positivity or last follow-up (drop-out, drug switch) or administrative censoring (12 months).

Here, the data are collected only at certain monitoring fixed timepoints (e.g. M0, M1, M3, M6). Thus, the occurrence of the event of interest is only known to lie in some interval. This kind of data is called interval-censored data.

It is worth noting that since the monitoring is regular, we may consider for a simplified modelling purpose that the time to first positivity of ADA status is right censored using the midpoint imputation.

Candidate Variables for prediction and selection

Genetic data

Genotyping information is obtained using InfiniumOmniExpress-24 Chip (Illumina). Data will be loaded into the Illumina BeadStudio software that converted fluorescence intensities into SNP genotypes. Samples with call rate below 97% will be excluded. SNP filters will be applied for excluding duplicate and Mendelian errors. The SNPs with call-rate lower than 90%, or MAF < 1% or showing significant deviation from Hardy-Weinberg Equilibrium (p-value $\leq 10^{-7}$) will be removed.

We will perform a statistical imputation of classical human leukocyte antigen (HLA) alleles using currently available HLA imputation packages.

A principal component analysis will be performed and we will use a subset of the top principal components (PCs) as confounding covariates in the survival model.

We will perform a gene prioritization based on biological information and select a set of susceptibility gene (with associated SNPs) candidates (cf ANNEX).

Bio-clinical data

Bio-clinical variables will be considered as either confounding variable or explanatory variables (influencing ADA occurrence) and will be entered in the final predictive model in different ways. In practice,

- We will adjust for the type of disease (RA, MS, IBD) and type of BP treatment.

- Candidate bio-clinical variables (that will be screened selected in a pre-selection process) are the following: disease duration at therapy start, age at start of the therapy, sex, Body Mass Index at baseline, dose of treatment, frequency of treatment, route of delivery, previous treatment, medical history (infectious mononucleosis, other autoimmune diseases, neoplasms, surgery and infections in the last year before therapy start), current comorbidities (other autoimmune/inflammatory diseases, allergies), family history of autoimmune diseases, concomitant medication (such as hormonal contraception, vitamin D supplementation, antibiotics, immunosuppressants, intravenous immunoglobulins), vaccinations during the last year before therapy start and during the study, early adverse events during the first BP injection or during the first month of therapy, last pregnancy and last breastfeeding, tobacco consumption.

Data collection and management

Patient clinical, demographic and epidemiological data were collected during the ABIRISK prospective studies ABI-MS-P01, ABI-RA-P01, ABI-IBD-P01 into a disease-specific eCRF. Serum and blood DNA samples were collected during the study visits and stored in local biobanks until delivery to the central labs that performed the assays.

ADA assays on the serum samples were performed in specific central ABIRISK laboratories for each BP as listed in the “Outcome definitions” paragraph.

Extraction of DNA was performed according to the SOP in the clinical immunology lab of the Kremlin-Bicêtre hospital. The genetic analysis was performed by hybridization of DNA to an Omniexpress chip (Illumina) at the P3S genotyping platform of the University Pierre and Marie Curie (Paris). All the data except the genotyping were loaded in the ABIRISK TranSMART database.

Validation procedures (internal validation)

Out-of-bag subjects will be used to obtain predictive accuracy estimation.

Data analysis

Essential analyses

The first aim of the study is to design a statistical tool for predicting the occurrence of ADAs in the first year of treatment taking into account for BPs and autoimmune disorders (MS, RA and IBD).
The second aim is to select bio-clinical markers associated with the occurrence of ADAs.

Basic descriptive statistics

Study participants will be described by baseline characteristics per disease (and per BP). Baseline characteristics will be compared between diseases (and BPs) using the chi-square test, Fisher's exact test, t-test or the Mann-Whitney test (depending on the nature of the variable and the sample size) when appropriate.

Univariate analysis for the bio-clinical variables

Taking into account the interval-censored nature of the data, the cumulative incidence of ADA occurrence (by BP, disease) will be estimated using the non-parametric maximum likelihood estimate for the distribution from interval censored data using the self-consistent estimator of Turnbull's algorithm (Sun). The 95% confidence intervals (95% CIs) will be obtained using a modified bootstrap method. For the univariate analyses, we will used logrank tests adapted for interval-censored data.

Basic multivariate analysis for the bio-clinical variables

From the univariate analysis and taking into account for multiple comparisons, we will select a set of candidate variables for a FDR of 5%. Then, we will fit a Cox proportional hazards model via a multiple imputation strategy for the unobserved survival times as proposed by Pan. Hazard ratios (HRs) and their 95% CIs will be reported.
For the final predictive modeling, we will consider as candidate bio-clinical variables: disease, drugs and variables that are significant ($p<5\%$) in the multivariate Cox model.

Predictive modeling

The outcome of interest is the time to first positive ADA which is interval-censored. Moreover, we are dealing with a mixed population of immune-reactive and immune-tolerant patients where the immune-tolerant ones will not experience ADA in the long term follow-up (12 months window interval).

For modeling the time-to-ADA occurrence survival distribution, we will consider a modified bagging procedure with a tree-structured improper survival model as proposed by C. Mbogning and P. Broët.
We will consider the bagging strategy to predict the cumulative hazard of a given individual using as basic learner the improper survival tree. The individual cumulative hazard function of each improper survival tree within the forest are aggregated to form a bagged cumulative hazard function.
Selection of genetic variables associated with the occurrence of ADAs will be done using the importance variable criteria previously developed by C. Mbogning and P. Broët.

General considerations for data analyses

Statistical language/software R will be used together with additional R packages

4. STUDY MANAGEMENT

Ethical approval and subject consent

Ethical approval and subject consent is obtained locally in each country where patients were recruited.

The current analysis will use anonymized data from the ABIRISK database.

Study reporting and publications

Data custodian and people involved in the collection of the data will participate to the publication and be co-author of these publications.

The results of these analyses will be reported to WP4 leaders and data providers. Interpretation of the results will be done collegially. A manuscript will be written and submitted for publication.

<u>Authorship (according to the ABIRISK communication policy):</u>

All investigators directly involved in a given piece of research, as mentioned in ABIRISK final Full Project (FPP), as well as other researchers demonstrating a significant involvement in the research are eligible to co-author the related scientific paper. Since WPL and WPCoLs in each WP are deeply involved in the scientific definition of ABIRISK objectives and methods, it is envisaged that they will be eligible to co-author all papers issued from their WP. Similarly, the coordinators of ABIRISK will be eligible to be included in the authorship of all papers issued from ABIRISK research projects. However it should be noted that they should also fulfil the criteria for authorship as outlined below.

Final authorship will require the fulfilment of the Uniform Requirements for Authorship and Contributorship from the International Committee of Medical Journal Editors (www.icmje.org): “Authorship credit should be based on 1) substantial contributions to conception and design, acquisition of data, or analysis and interpretation of data; 2) drafting the article or revising it critically for important intellectual content; and 3) final approval of the version to be published. Authors should meet conditions 1, 2, and 3.”

5. REFERENCES

Bartelds GM, Krieckaert CLM, Nurmohamed MT, Schouwenburg PA van, Lems WF, Twisk JWR, et al. 2011. Development of antidrug antibodies against adalimumab and association with disease activity and treatment failure during long-term follow-up. JAMA J. Am. Med. Assoc. 305:1460–1468; doi:10.1001/jama.2011.406.

Buck D, Cepok S, Hoffmann S, Grummel V, Jochim A, Berthele A, et al. 2011. Influence of the HLA-DRB1 genotype on antibody development to interferon beta in multiple sclerosis. Arch. Neurol. 68:480–487; doi:10.1001/archneurol.2011.65.

Hesse D, Sørensen PS. 2007. Using measurements of neutralizing antibodies: the challenge of IFN-β therapy. Eur. J. Neurol. 14:850–859; doi:10.1111/j.1468-1331.2007.01769.x.

Hoffmann S, Cepok S, Grummel V, Lehmann-Horn K, Hackermüller J, Hackermueller J, et al. 2008. HLA-DRB1*0401 and HLA-DRB1*0408 are strongly associated with the development of antibodies against interferon-beta therapy in multiple sclerosis. Am. J. Hum. Genet. 83:219–227; doi:10.1016/j.ajhg.2008.07.006.

Link J, Lundkvist Ryner M, Fink K, Hermanrud C, Lima I, Brynedal B, Kockum I, Hillert J, Fogdell-Hahn A. 2014. Human leukocyte antigen genes and interferon beta preparations influence risk of developing neutralizing anti-drug antibodies in multiple sclerosis. PLoS One. 2014 Mar 7;9(3):e90479. doi: 10.1371/journal.pone.0090479.

Núñez C, Cénit MC, Alvarez-Lafuente R, Río J, Fernández-Arquero M, Arroyo R, Montalbán X, Fernández O, Oliver-Martos B, Leyva L, Comabella M, Urcelay E. 2014. HLA alleles as biomarkers of high-titre neutralising antibodies to interferon-β therapy in multiple sclerosis. J Med Genet. 2014 Apr 19. doi: 10.1136/jmedgenet-2014-102348.

Lee LYW, Sanderson JD, Irving PM. 2012. Anti-infliximab antibodies in inflammatory bowel disease: prevalence, infusion reactions, immunosuppression and response, a meta-analysis. Eur. J. Gastroenterol. Hepatol. 24:1078–1085; doi:10.1097/MEG.0b013e32835558cf.

Lundkvist M, Greiner E, Hillert J, Fogdell-Hahn A. 2010. Multiple sclerosis patients lacking oligoclonal bands in the cerebrospinal fluid are less likely to develop neutralizing antibodies against interferon beta. Mult. Scler. 16:796–800; doi:10.1177/1352458510373112.

Hedström A, Alfredsson L, Lundkvist Ryner M, Fogdell-Hahn A, Hillert J, Olsson T. 2013. Smokers run increased risk of developing anti-natalizumab antibodies. Mult Scler. 2013 Dec 5.

Hedström AK, Ryner M, Fink K, Fogdell-Hahn A, Alfredsson L, Olsson T, Hillert J. 2014. Smoking and risk of treatment-induced neutralizing antibodies to interferon β-1a. Mult Scler. 2014 Apr;20(4):445-50. doi: 10.1177/1352458513498635.

Mbogning, C. and Broët, P. (2016). Bagging survival tree procedure for variable selection and prediction in the presence of nonsusceptible patients. BMC bioinformatics , 17(1):230.

Mbogning, C. and Broet, P. (2017).1.0. iBST: improper Bagging Survival Tree R package version

Sorensen PS, Koch-Henriksen N, Ross C, Clemmesen KM, Bendtzen K. 2005. Appearance and disappearance of neutralizing antibodies during interferon-beta therapy. Neurology 65:33–39; doi:10.1212/01.WNL.0000166049.51502.6A.

Sun, J The Statistical Analysis of Interval-censored Failure Time Data (book) Wiley

6. ANNEX – Genes of interest

The specific genes that will be analysed (using the information of the corresponding tag SNPs) are:

HLA alleles (HLA-DRB1, HLA-A, HLA-B), Vitamin D metabolism and response genes (VDR, RXRB, DBP/GC, CYP2R1, CYP27A1, CYP27B1, CYP24A1, DHCR7, IL10, IL12B, IL4R), IFNbeta response genes (IFNB1, IFNaR1, IFNaR2, MxA, STAT1, STAT3, IRF-1, IRF-5, IRF-7, IRF-8, TRAILR1, ISG15, USP18, CXCL10), innate immunity receptor genes (TLR2, TLR3, TLR4, TLR6, TLR7, TLR8, TLR9, NOD1, NOD2), costimulatory molecule genes (CD37, CD40, CD80, CD86, VCAM-1, CD58, CTLA4, CLECL1, ICOSLG), sex hormone related genes (ESR1, ESR2, CYP17A1, ER response genes like SNPs in ERE in AID, androgen receptor, etc.), other MS, RA and IBD susceptibility genes (IL2RA, CD6, TNFRSF1A, TNFRSF14, TNFSF14, CLEC16A, CIITA, CXCR5, IL12RB1, IL22RA2, IL27, PTGER4, TYK2), other B-cell and ADA-related genes (IL7, IL7R, AIRE, FcgRIIa, FcgRIIIa, FcgRIIb, FcgRIIIb, FcgRIIc, BAFF, APRIL, TNFalpha, HMOX-1, MAPK9, PCGF2, DOCK2, CD44, CSFR1, HSP90B1, IGSF2, ALOX5AP, MAP2K4, PTPRN2, PTPRM, PTPRE, MSR1, TNFRSF21, TNFSF8, CD19, CD9, CD21, CD35, ICOSL, CXCL13, PRDM1).
