# Supplementary material for: Clinicogenomic factors of biotherapy immunogenicity in autoimmune disease: A prospective multicohort study of the ABIRISK consortium
Source: PLoS Med. 2020 Oct 30;17(10):e1003348. doi: 10.1371/journal.pmed.1003348 (PMC7598520; doi:10.1371/journal.pmed.1003348)
Supplement: S1 Table — ACPA, anti-citrullinated peptide antibodies; BP, biopharmaceutical product; CIS, Clinically Isolated Syndrome; DAS28, disease activity score 28; EDSS, expanded disability status scale; IBD, inflammatory bowel disease; IQR, interquartile range; MS, multiple sclerosis; RA, rheumatoid arthritis; RF, rheumatoid factor; RRMS, relapsing remitting multiple sclerosis. (PDF) [file pmed.1003348.s003.pdf]

S1 Table. Clinical characteristics at baseline in BP-treated patients stratified by disease cohort

Characteristics	MS<br>(n =147)	RA<br>(n =229)	IBD<br>(n=184)
Disease duration, median (IQR), years	0.85 (2.28)	3.17 (7.58)	3.69 (10.37)
Family history of the same disease, n (%)	7 (4.76)	41 (17.9)	33 (17.9)
Vaccines during the last year, n (%)	7 (4.76)	84 (36.68)	28 (15.21)
Disease type, n (%)			
CIS	45 (30.6)	-	-
RRMS	101 (68.7)	-	-
Secondary progressive	1 (0.7)	-	-
EDSS, median (IQR)	1.75 (1.00)	-	-
Number of relapses in the last two years, median (IQR)	1.00 (1.00)	-	-
Disease type, n (%)			
Crohn's disease	-	-	148 (80.4)
Ulcerative colitis	-	-	36 (19.6)
Mayo score, median (IQR)	-	-	6.00 (5.00)
Harvey-Bradshaw Index, median (IQR)	-	-	5.00 (5.00)
Disease location, n (%)			
Colonic	-	-	27 (18.4)
Ileal	-	-	66 (44.9)
Ileocolonic	-	-	53 (36.1)
Isolated Upper Disease	-	-	1 (0.6)
Disease behavior, n (%)			
Non-stricturing, non-penetrating	-	-	68 (46.3)
Penetrating	-	-	28 (19.0)
Stricturing	-	-	51 (34.7)
RF positivity, n (%)	-	149 (68.0)	-
ACPA positivity, n (%)	-	152 (70.0)	-
DAS28, median (IQR)	-	4.55 (1.68)	-

BP, Biopharmaceutical product; MS, multiple sclerosis; RA, rheumatoid arthritis; IBD, inflammatory bowel disease; IQR, interquartile range; CIS, clinically isolated syndrome; RRMS, relapsing remitting multiple sclerosis; EDSS, expanded disability status scale; RF, rheumatoid factor; ACPA, anti-citrullinated peptide antibodies; DAS28, disease activity score 28.
