# Supplementary material for: Clinicogenomic factors of biotherapy immunogenicity in autoimmune disease: A prospective multicohort study of the ABIRISK consortium
Source: PLoS Med. 2020 Oct 30;17(10):e1003348. doi: 10.1371/journal.pmed.1003348 (PMC7598520; doi:10.1371/journal.pmed.1003348)
Supplement: S2 Table — MedDRA, medical dictionary for regulatory activities; MS, multiple sclerosis; NA, nonapplicable; RA, rheumatoid arthritis; UC, ulcerative colitis; WHODrug, World Health Organization drug dictionary. (DOCX) [file pmed.1003348.s004.docx]

|  | **MedDRA code** | | **WHODrug code** | **N** | **NAs** |
| --- | --- | --- | --- | --- | --- |
| **Age** | NA | NA | | 560 | 0 |
| **Sex** | NA | NA | | 560 | 0 |
| **Smoke** | NA | NA | | 555 | 5 |
| **BMI** | NA | NA | | 551 | 9 |
| **Family history same disease** | 10039073 (RA)  10011401 (Crohn)  10009900 (UC)  10028245 (MS) | NA | | 560 | 0 |
| **Past infections** | 10021879 | NA | | 560 | 0 |
| **Hypertension** | 10020772 | NA | | 560 | 0 |
| **Metabolic disease** | 10027433 | NA | | 560 | 0 |
| **Neoplastic disease** | 10029104 | NA | | 560 | 0 |
| **Nervous system disease** | 10029205 | NA | | 560 | 0 |
| **Respiratory disease** | 10038738 | NA | | 560 | 0 |
| **Past immunosuppressants** | NA | L04 | | 544 | 16 |
| **Past corticosteroids** | NA | H02 | | 544 | 16 |
| **Vaccines last year** | NA | J07 | | 560 | 0 |
| **Infections (time-dependent)** | 10021879 | NA | | 560 | 0 |
| **Immunosuppressants (time-dep.)** | NA | L04 | | 560 | 0 |
| **Analgesics (time-dependent)** | NA | N02 | | 560 | 0 |
| **Antibiotics (time-dependent)** | NA | J01 | | 560 | 0 |
| **Corticosteroids (time-dependent)** | NA | H02 | | 560 | 0 |
| **Vaccines (time-dependent)** | NA | J07 | | 560 | 0 |
| **Vitamins (time-dependent)** | NA | A11 | | 560 | 0 |
| **Drugs for acid related disorders (time dependent)** | NA | A02 | | 560 | 0 |

**S2 Table. Missing data, MedDRA and WHODrug codes for the demographics and clinical variables.**

MedDRA, medical dictionary for regulatory activities; WHODrug, world health organization drug dictionnary; NA, non applicable; RA, rheumatoid arthritis; UC, ulcerative colitis; MS, multiple sclerosis.
