# Supplementary figures and images for: Clinicogenomic factors of biotherapy immunogenicity in autoimmune disease: A prospective multicohort study of the ABIRISK consortium
Source: PLoS Med. 2020 Oct 30;17(10):e1003348. doi: 10.1371/journal.pmed.1003348 (PMC7598520; doi:10.1371/journal.pmed.1003348)

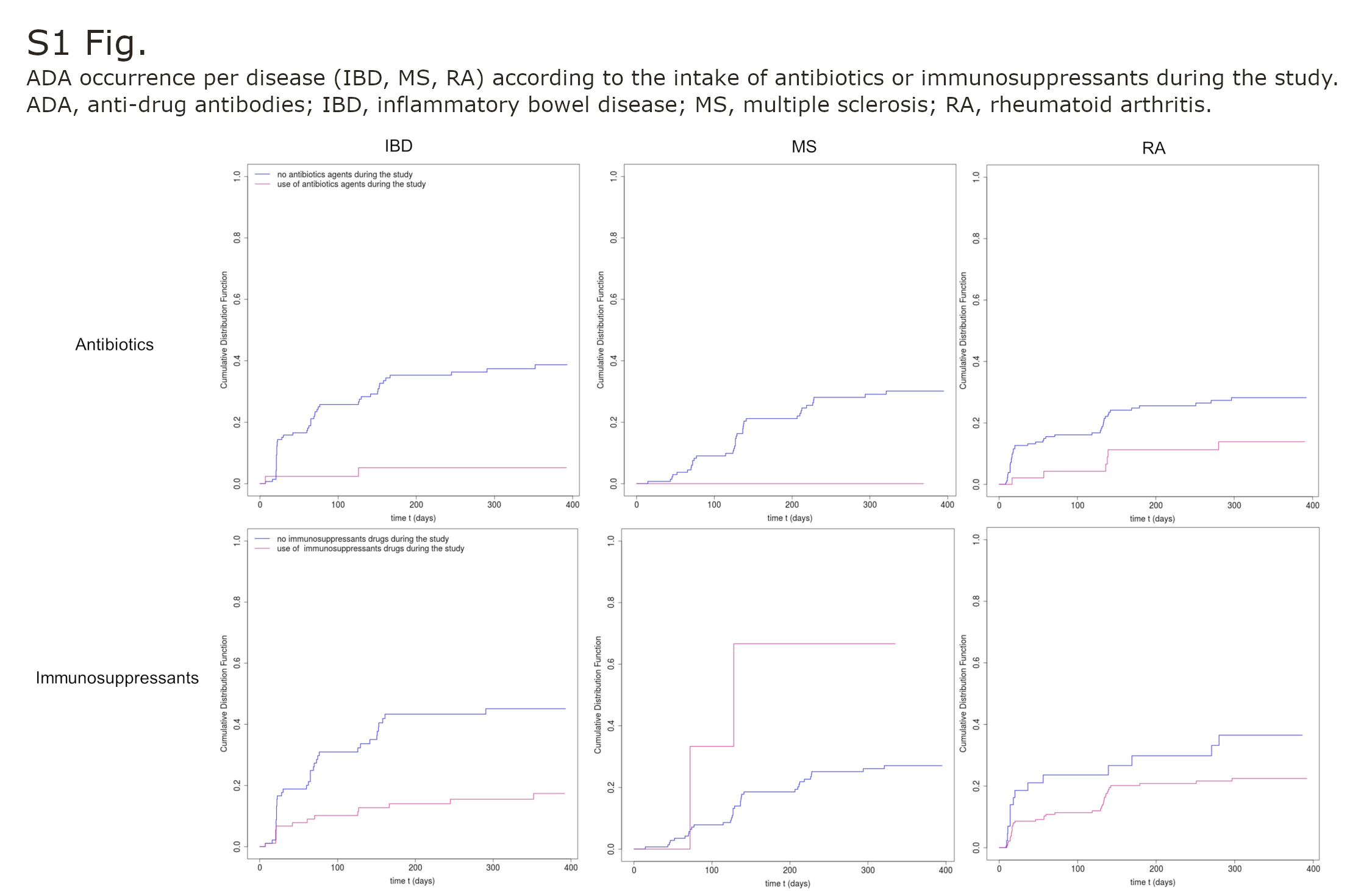

Supplement: S1 Fig — ADA, antidrug antibody; IBD, inflammatory bowel disease; MS, multiple sclerosis; RA, rheumatoid arthritis. (TIF) [file pmed.1003348.s006.tif]

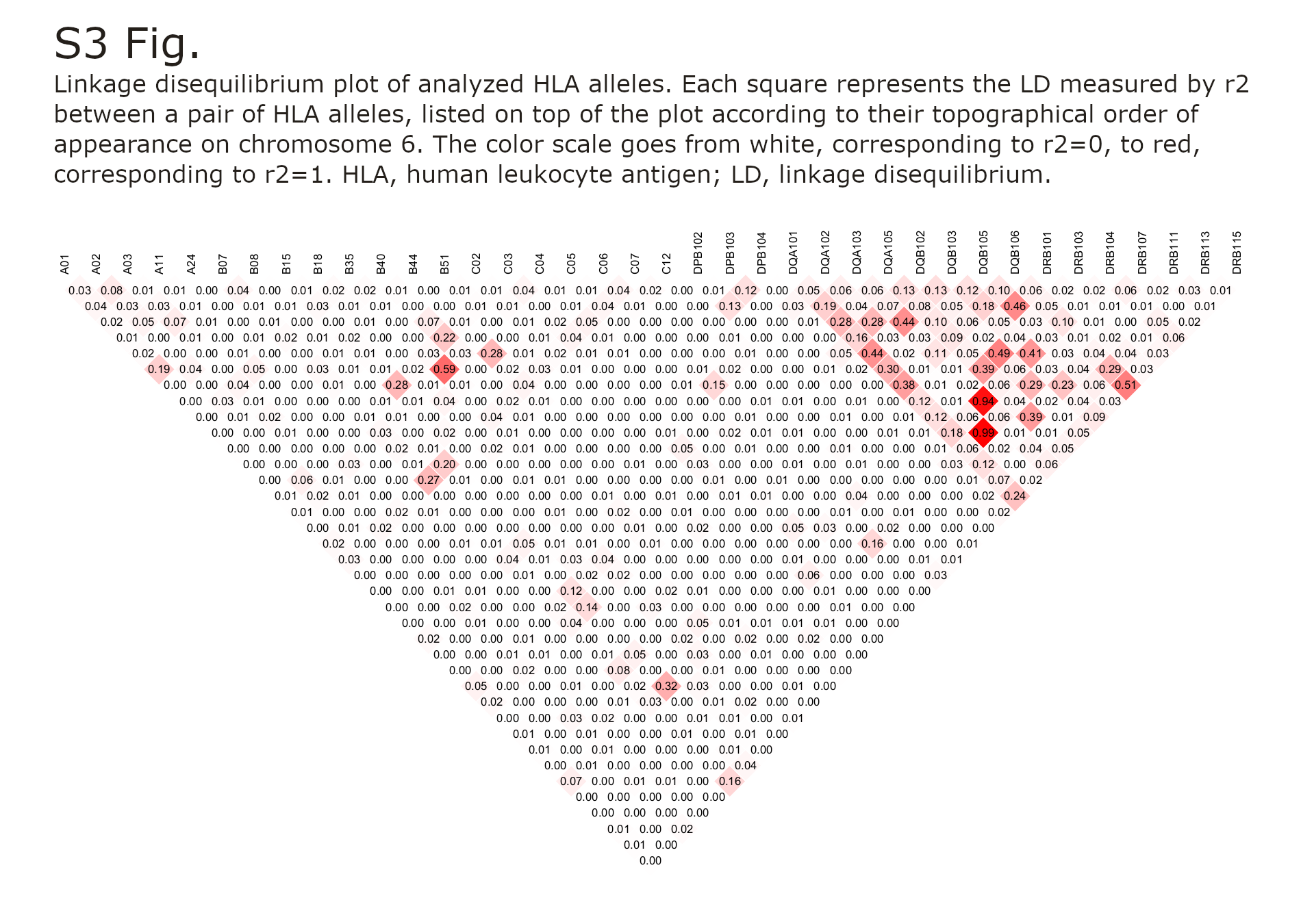

Supplement: S3 Fig — Each square represents the LD measured by r2 between a pair of HLA alleles, listed on top of the plot according to their topographical order of appearance on chromosome 6. The color scale goes from white, corresponding to r2 = 0, to red, corresponding to r2 = 1. HLA, Human Leukocyte Antigen; LD, linkage disequilibrium. (TIF) [file pmed.1003348.s008.tif]
