# Supplementary material for: Clinicogenomic factors of biotherapy immunogenicity in autoimmune disease: A prospective multicohort study of the ABIRISK consortium
Source: PLoS Med. 2020 Oct 30;17(10):e1003348. doi: 10.1371/journal.pmed.1003348 (PMC7598520; doi:10.1371/journal.pmed.1003348)

S2 Fig

GWAS results (Manhattan plot). Genomic coordinates on 23 chromosomes are displayed on the x-axis, the negative logarithm of the association P-value with ADA occurrence for each SNP is displayed on the y-axis. The blue horizontal dotted line represents the threshold of significance with a 20% FDR and the red dots are the SNPs above the threshold.
GWAS, genome-wide association study; SNP, single nucleotide polymorphism.

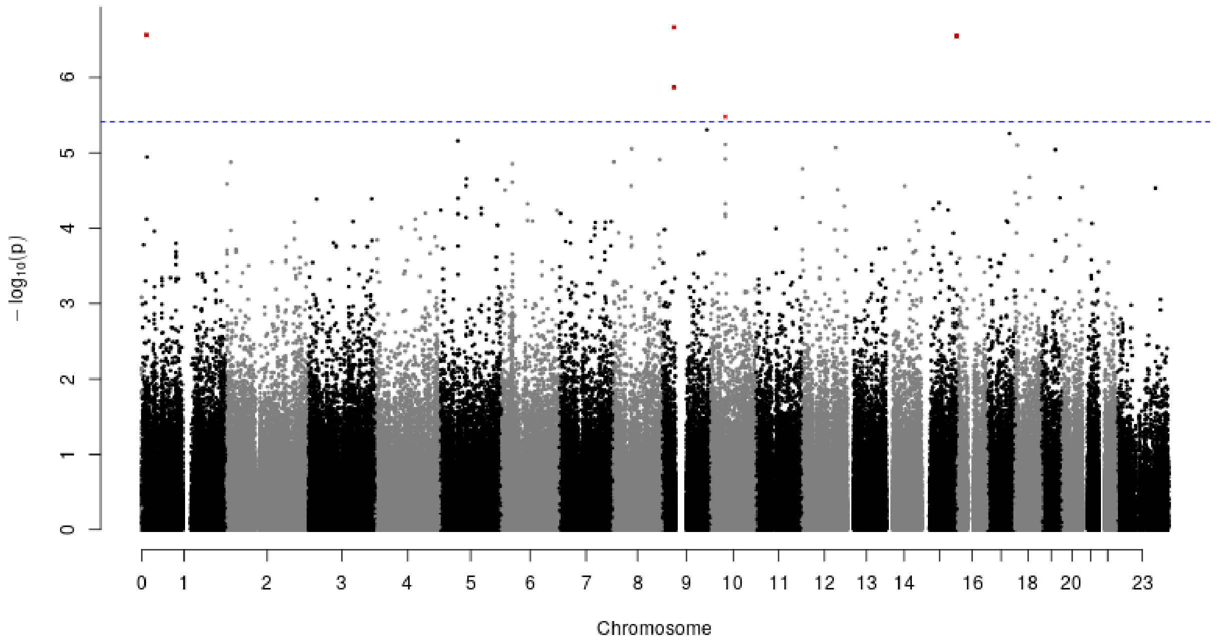

Supplement: S2 Fig — Genomic coordinates on 23 chromosomes are displayed on the x-axis, and the negative logarithm of the association p-value with ADA occurrence for each SNP is displayed on the y-axis. The blue horizontal dotted line represents the threshold of significance with a 20% FDR, and the red dots are the SNPs above the threshold. ADA, antidrug antibody; FDR, false discovery rate; GWAS, genome-wide association study; SNP, Single-Nucleotide Polymorphism. (PDF) [file pmed.1003348.s007.pdf]
